# Linking Stone Mineral Composition to 24-Hour Urinary Metabolic Profiles: An Episode-Based Cohort Study

**DOI:** 10.3390/metabo16100711

**Published:** 2026-09-24

**Authors:** Shiwei Sun, Jiang Liu, Yi Qiao, Dong Wang, Lin Ma, Hongjun Li, He Xiao

**Affiliations:** Department of Urology, Peking Union Medical College Hospital, Chinese Academy of Medical Sciences & Peking Union Medical College, Beijing 100730, China; sunshiwei@student.pumc.edu.cn (S.S.); b2024001210@student.pumc.edu.cn (J.L.); qiaoyi@pumch.cn (Y.Q.); wangdong53@pumch.cn (D.W.)

**Keywords:** urolithiasis, stone composition, 24 h urine, calcium oxalate, carbonate apatite, metabolic phenotyping

## Abstract

**Highlights:**

**What are the main findings?**

- Urine pH was the clearest metabolic marker: higher pH was associated with carbonate apatite, whereas lower pH was associated with anhydrous uric acid.
- Low urinary citrate became more common as stone composition shifted from calcium oxalate towards calcium phosphate.

**What are the implications of the main findings?**

- A metabolic result should be interpreted alongside the complete stone composition, particularly for mixed calcium oxalate–calcium phosphate stones.
- Stone analysis and 24 h urine testing provide different but complementary information for personalised stone prevention.

**Abstract:**

Background/Objectives: Stone composition reflects the urinary environment in which calculi form, whereas 24 h urine testing identifies modifiable lithogenic abnormalities. This study examined component-specific urinary metabolic profiles and evaluated whether routine clinical and urinary variables could distinguish common stone minerals. Methods: This retrospective study included 256 patients undergoing 297 stone surgeries, consolidated into 256 treatment episodes. Fourier-transform infrared spectroscopy identified stone minerals, which were linked to preoperative 24 h urine profiles. Calcium oxalate monohydrate (COM), calcium oxalate dihydrate (COD), carbonate apatite, and anhydrous uric acid were evaluated using multivariable logistic regression. Mutually exclusive calcium oxalate–calcium phosphate phenotypes, nested cross-validated prediction models, and unsupervised clustering were also examined. Results: COM, COD, carbonate apatite, and anhydrous uric acid were present in 78.5%, 73.0%, 39.5%, and 9.8% of episodes, respectively. Higher urine pH was associated with carbonate apatite (odds ratio [OR] per standard deviation, 1.78; 95% confidence interval [CI], 1.33–2.38), whereas lower pH was associated with anhydrous uric acid (OR, 0.26; 95% CI, 0.11–0.58). Higher urinary citrate was associated with COM, but phenotype analysis showed that hypocitraturia increased from 45.3% in calcium oxalate-only episodes to 60.7% in mixed calcium oxalate–calcium phosphate episodes and 89.5% in calcium phosphate episodes without calcium oxalate. Lower urine volume was associated with COD. Cross-validated areas under the curve ranged from 0.654 to 0.712, and metabolic clusters showed substantial mineral overlap. Conclusions: Urine pH provided the clearest component-specific signal, whereas citrate associations depended on accompanying minerals. Routine 24 h urine testing complemented but did not replace direct stone analysis.

## 1. Introduction

Urolithiasis is common, and recurrence remains frequent [1]. Endourological treatment can clear most visible stones [2,3,4,5], but it does not correct the urinary conditions in which they formed [6,7]. Preventive care therefore requires attention to both the mineral recovered at surgery and the metabolic environment that produced it.

A stone records the crystalline phases accumulated during its growth [8], whereas urine testing reflects a much shorter and potentially variable interval. It can identify the current urinary environment relevant to stone risk but cannot reconstruct the full temporal sequence in which the stone developed [9]. Both tests are recommended for metabolic evaluation and secondary prevention, although in practice their findings are often considered separately [6,7].

Clinical studies have identified several composition-specific urinary patterns. Compared with calcium oxalate (CaOx) stone formers, patients with calcium phosphate (CaP) stones generally have higher urine pH and lower urinary citrate, whereas uric acid stones are most consistently associated with acidic urine [10,11,12]. For calcium oxalate monohydrate (COM) and calcium oxalate dihydrate (COD), the evidence is less consistent: an early multicentre study linked COD to higher urinary calcium and lower citrate, whereas a recent cohort found little overall separation between COM and COD using conventional 24 h urine measurements [13,14]. Previous studies have also used different mineral definitions, ranging from predominant stone type to the presence of a component [12,14]. It therefore remains uncertain whether an observed association belongs to the mineral of interest or partly reflects other minerals in its comparison group.

An earlier study from our group related 24 h urine profiles to anatomical stone location [15]. The present study addresses a different question: which urinary features are associated with the minerals actually recovered from the stone? This distinction is clinically relevant because metabolic findings are interpreted differently across uric acid, CaOx and CaP stones. Acidic urine, for example, supports urinary alkalinisation for uric acid stones, whereas a CaP-rich composition makes monitoring the pH response to alkali particularly important [6,7,10,11]. Anatomical location and mineral composition therefore provide complementary information, but composition more directly links the urine profile to the crystalline endpoint of stone formation.

This study aimed to characterise the independent associations between routinely measured preoperative urinary variables and FTIR-defined COM, COD, carbonate apatite and anhydrous uric acid in an episode-based surgical cohort, with staged procedures for the same stone burden consolidated into treatment episodes. A further objective was to determine whether mutually exclusive CaOx–CaP phenotypes could clarify citrate associations potentially influenced by mineral coexistence. Prediction modelling and unsupervised clustering were secondary analyses.

## 2. Materials and Methods

### 2.1. Study Population and Treatment Episodes

This retrospective study included all operations for urinary tract stones performed by one surgical team between May 2024 and May 2026. Eligible procedures involved renal, ureteric, bladder or multisite stones. The included procedures were flexible ureteroscopy, including retrograde intrarenal surgery (RIRS), as well as semirigid ureteroscopic lithotripsy, cystoscopic lithotripsy and percutaneous nephrolithotomy. The full cohort comprised 443 patients and 510 operations and was used to describe the spectrum of stone composition encountered in surgical practice. The metabolic analysis required a complete preoperative 24 h urine evaluation. After 213 operations without such an evaluation were excluded, 297 operations in 256 patients remained (Figure 1a). The study was conducted in accordance with the Declaration of Helsinki and was approved by the Institutional Review Board of Peking Union Medical College Hospital (Approval Code: K23C2872; Approval Date: 12 September 2023). Written informed consent was obtained from all patients.

**Figure 1:**
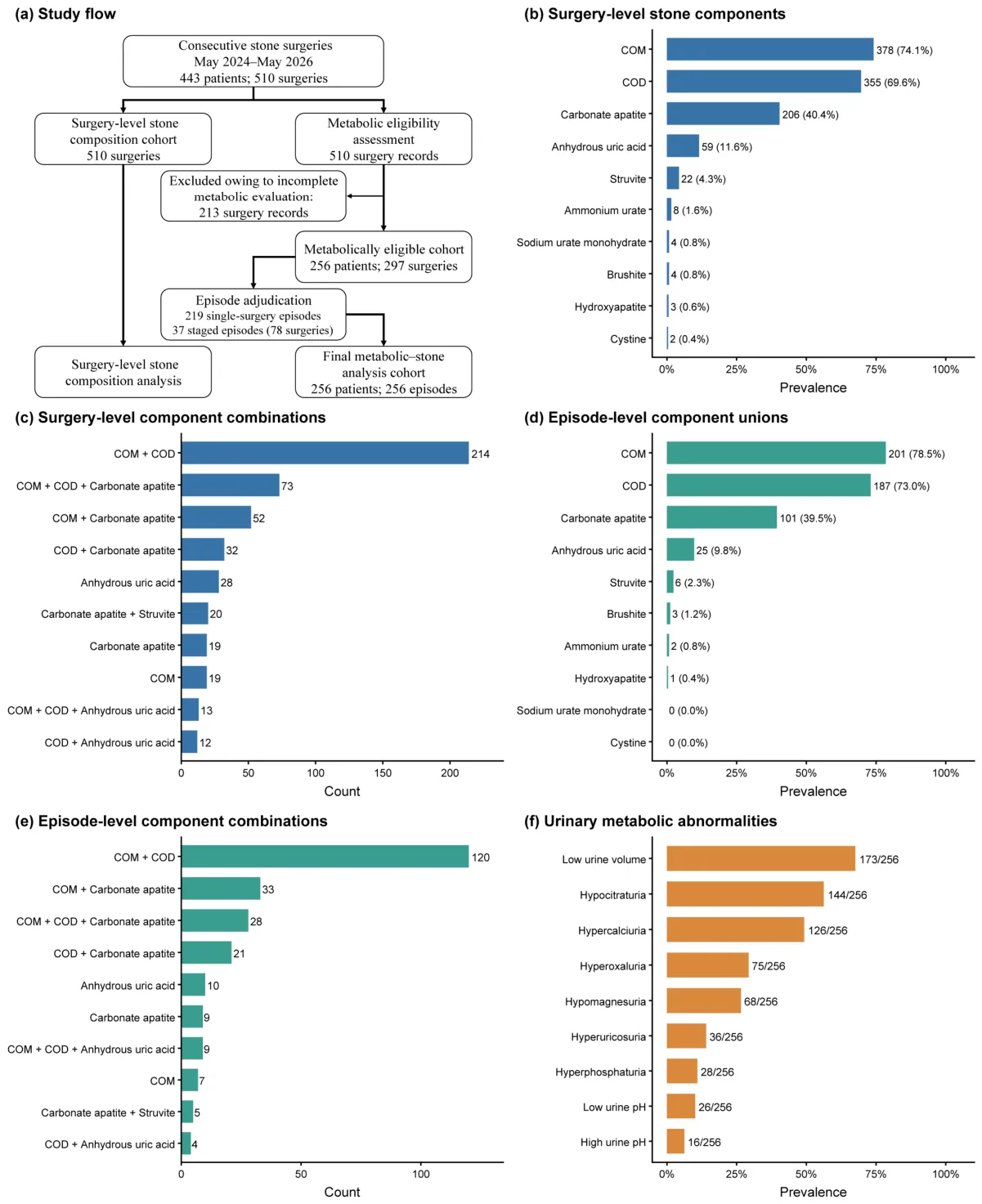
Study flow, stone-component profiles and urinary metabolic abnormalities. ((**a**) Study flow from 510 consecutive stone surgeries in 443 patients to 297 metabolically eligible surgeries and 256 adjudicated treatment episodes; (**b**) prevalence of individual stone components among 510 surgeries; (**c**) distribution of the ten most frequent component combinations among 510 surgeries; (**d**) prevalence of stone components after component reports were combined within each of the 256 treatment episodes; (**e**) distribution of the ten most frequent component combinations among 256 treatment episodes; (**f**) prevalence of nine urinary metabolic abnormalities among 256 treatment episodes).

The unit of metabolic analysis was the treatment episode rather than the individual operation. For patients with more than one operation, the study team reviewed the interval between procedures, laterality, anatomical site and stone burden before the first operation. Procedures judged to represent staged treatment of the same burden were combined; uncertain cases were resolved by further chart review and consensus. Of the 256 patients, 219 underwent one eligible operation and 37 underwent 78 staged operations. Within a staged episode, composition was defined by the union of reliable reports and stone burden by the total burden before the first procedure. The final analysis contained 256 independent episodes.

### 2.2. Clinical Assessment and FTIR Stone Analysis

Demographic and clinical data included age, sex, body mass index (BMI), hypertension, diabetes mellitus and estimated glomerular filtration rate (eGFR) calculated using the 2021 Chronic Kidney Disease Epidemiology Collaboration (CKD-EPI) equation. Stone characteristics comprised anatomical site, laterality, lower-calyx involvement, staghorn configuration, multiplicity, cumulative stone diameter (CSD) and computed tomography attenuation. Operative variables included ureteric stenting, procedure duration, length of stay and stone-free status. For staged treatment, anatomical features and final clearance were determined for the episode as a whole.

Postoperative stone specimens were washed, dried and ground to a fine powder. Stone composition was determined by Fourier-transform infrared spectroscopy using the KBr-pellet transmission method on an LIIR-20 automated stone analysis system (Lanmode Scientific Instrument Co., Ltd., Tianjin, China). Spectra were processed using the system’s proprietary matching algorithm and subsequently reviewed by laboratory personnel. According to the system’s classification rule, stones in which a single component was estimated to account for at least 95% were operationally classified as pure; all others were classified as mixed. Routine clinical reports listed the identified components without reporting their continuous percentages. COM, COD, carbonate apatite and anhydrous uric acid were prespecified for component-specific analyses, while less common minerals remained in the descriptive composition profile.

### 2.3. Preoperative Urine and Blood Sampling and Laboratory Measurements

Preoperative urine and blood sampling was undertaken 1–7 days before surgery. Patients maintained their usual diet and water intake during urine collection. Citrate preparations, thiazide diuretics and allopurinol were withheld under medical supervision, whereas antibacterial treatment, when clinically indicated, was not withheld for study purposes. Patients were instructed to avoid non-water beverages, unusually high fluid intake, strenuous exercise and sleep deprivation during collection, and women avoided collection during menstruation. Specimens visibly contaminated by blood, purulent material or genital secretions were not accepted.

Two consecutive 24-hour urine collections were obtained. At approximately 07:00 on day 1, an aliquot of the first-morning void was collected for urinalysis and urine pH measurement, and the remainder was discarded. The first timed collection then began and continued until approximately 07:00 on day 2, with the terminal void included in that collection. This specimen was used for routine 24 h urine biochemistry and was collected in a 5 L lidded polypropylene container containing one 10 g sachet of boric acid.

The second timed collection began immediately after the day-2 terminal void and continued until approximately 07:00 on day 3, again including the terminal void. This specimen was collected separately for measurement of oxalate, citrate and cystine, using a clean, dry, colourless 5 L polypropylene container containing 5 mL of diazolidinyl urea. At the end of each collection, the urine was thoroughly mixed, the total volume was recorded and an aliquot was submitted for analysis. Collection containers were refrigerated or kept in a cool place in accordance with the laboratory instructions. Foods rich in vitamin C and vitamin C supplements were avoided during the oxalate, citrate and cystine collection. Collection completeness was assessed from adherence to the timed collection protocol; no retrospective exclusion threshold based on urinary creatinine relative to body weight was imposed.

The urinary metabolic profile comprised first-morning spot urine pH, 24 h urine volume and 24 h excretion of calcium, oxalate, citrate, uric acid, magnesium, cystine, sodium, potassium, chloride, phosphorus, urea and creatinine. Urine pH was measured in the first-morning urine specimen on day 1 using automated reagent-strip reflectance photometry. Sodium, potassium and chloride were measured using ion-selective electrodes; creatinine by an enzymatic method; urea by a urease method; phosphorus by the phosphomolybdate ultraviolet method; calcium by the arsenazo III method; magnesium by the xylidyl blue method; and uric acid by the uricase method. Oxalate, citrate and cystine were measured by liquid chromatography–tandem mass spectrometry using an AB SCIEX API 4500 triple-quadrupole mass spectrometer (AB SCIEX, Framingham, MA, USA). Twenty-four-hour excretion was calculated from the measured concentration and recorded urine volume.

Hypocitraturia, hypercalciuria, hyperoxaluria, hypomagnesuria, hyperuricosuria, hyperphosphaturia, low urine volume, low urine pH and high urine pH were defined according to European Association of Urology (EAU) guidance [6,7]. Continuous measurements were used for composition–metabolism associations and prediction, whereas the guideline-based categories described the prevalence of clinically recognisable metabolic abnormalities.

Fasting peripheral venous blood was collected at approximately 07:00 on day 1, concurrently with the first-morning urine specimen. Serum creatinine was measured enzymatically and used to calculate eGFR. Secondary serum analyses included calcium, albumin-corrected calcium, phosphate, magnesium, uric acid, parathyroid hormone, 25-hydroxyvitamin D, total carbon dioxide, potassium, glucose, triglycerides, total cholesterol, high-density lipoprotein cholesterol and low-density lipoprotein cholesterol. Albumin was measured by the bromocresol green method; calcium by the arsenazo III method; phosphate by the phosphomolybdate ultraviolet method; magnesium by the xylidyl blue method; uric acid by the uricase method; total carbon dioxide by an enzymatic method; potassium by indirect ion-selective electrode measurement; glucose by the hexokinase method; triglycerides by the GPO-PAP method; total cholesterol by the CHOD-PAP method; and high- and low-density lipoprotein cholesterol by homogeneous enzymatic methods. Albumin-corrected calcium was calculated as measured calcium + 0.02 × (40 − serum albumin [g/L]). Parathyroid hormone was measured by electrochemiluminescence, whereas total 25-hydroxyvitamin D was calculated as the sum of 25-hydroxyvitamin D2 and D3 measured by liquid chromatography–tandem mass spectrometry on a SCIEX 4500MD system (SCIEX, Framingham, MA, USA).

Evidence of preoperative urinary tract infection included a positive urine culture, nitrite positivity or pyuria (≥10 white blood cells per high-power field [WBC/HPF]).

### 2.4. Mineral Outcomes and Secondary Phenotypes

Each principal mineral was analysed separately as present or absent, allowing a mixed episode to contribute to more than one outcome. The opposite citrate coefficients for COM and carbonate apatite prompted a comparison of five mutually exclusive phenotypes: CaOx without CaP, mixed CaOx–CaP, CaP without CaOx, uric acid-related stones without CaOx, and other compositions.

Infection-stone analyses used two complementary definitions. Stones containing carbonate apatite, struvite or ammonium urate were classified as infection-associated according to the EAU mineral classification [7]. Among these episodes, a separate subgroup was defined by the additional presence of preoperative evidence of urinary tract infection. The first definition reflects mineral composition, whereas the second indicates documented infection around the time of treatment.

### 2.5. Statistical Analysis

All statistical analyses were performed using R version 4.5.2 (R Foundation for Statistical Computing, Vienna, Austria). Continuous variables are reported as median [interquartile range] and categorical variables as number (percentage). Component-positive and component-negative groups were compared using the Wilcoxon rank-sum test, Pearson chi-square test or Fisher exact test, as appropriate; standardised mean differences were used to describe the size of group differences.

For the association analyses, candidate urinary variables were defined separately for each mineral outcome on biological grounds. Urine volume, calcium, oxalate, citrate and pH were evaluated for COM and COD; pH, citrate, calcium and phosphorus for carbonate apatite; and pH, uric acid and volume for anhydrous uric acid. Each candidate was first examined using univariable logistic regression. Variables with *p* < 0.05 entered the corresponding multivariable logistic model and were removed sequentially, beginning with the largest *p* value, until all retained urinary variables had *p* < 0.10. This procedure was used to obtain parsimonious component-specific models while limiting the number of urinary terms relative to the less frequent outcome category.

Age, sex, BMI and eGFR were included in every multivariable association model and were not subject to variable selection. These covariates were specified because age, sex, adiposity and kidney function may influence both urine chemistry and the distribution of stone minerals [9,16,17]. Continuous predictors were standardised so that odds ratios (ORs) represent the change in odds associated with a one-standard-deviation increase.

Variable-selection stability was assessed by repeating the complete univariable-screening and backward-elimination procedure in 500 bootstrap samples. Selection frequency was defined as the proportion of valid bootstrap samples in which a urinary variable remained in the final model; the consistency of coefficient direction was also examined. Bootstrap results were used to describe the sample dependence of variable selection and did not replace the estimates obtained from the original cohort. Model convergence, collinearity, fitted probabilities and the number of observations in the less frequent outcome category relative to the number of fitted parameters were also examined.

The apparently divergent associations of urinary citrate with calcium oxalate and calcium phosphate components were further examined using mutually exclusive mineral phenotypes. Differences in continuous and categorical metabolic variables across these phenotypes were evaluated using the Kruskal–Wallis test and Fisher’s exact test, respectively. Infection-stone analyses compared the mineral-based definition recommended by the European Association of Urology with a definition additionally requiring preoperative evidence of urinary tract infection.

Serum measurements were evaluated separately for each stone component using univariable logistic regression and available cases, without imputation. Because the urinary models addressed a prespecified primary set of component-specific associations, no false-discovery-rate correction was applied to their screening *p* values; these findings were interpreted using adjusted ORs, 95% confidence intervals and bootstrap selection frequencies. By contrast, the serum analysis involved 14 parallel secondary comparisons for each component, and Benjamini–Hochberg false-discovery-rate correction was therefore applied separately within each component.

Prediction analyses compared AIC-based stepwise logistic regression, ridge regression, least absolute shrinkage and selection operator (LASSO), elastic net, random forest and extreme gradient boosting (XGBoost). Stepwise logistic regression represented conventional subset selection; ridge, LASSO and elastic net allowed linear shrinkage and variable selection in the presence of correlated predictors; and the tree-based models allowed nonlinear associations and interactions.

Two predictor representations were considered. The continuous-variable library comprised 50 demographic, clinical, stone, infection, urinary and serum predictors, including 14 continuous urinary measurements and 14 serum measurements. The alternative flag-dominant library comprised 51 predictors, replacing urinary measurements with guideline-defined abnormality indicators where available while retaining six urinary measurements without corresponding flags. Four training-fold screening strategies were compared: retaining all candidate predictors, or retaining predictors with univariable *p* values below 0.05, 0.10 or 0.20.

The outer validation procedure comprised two repeats of stratified five-fold cross-validation. All preprocessing, missing-data handling and univariable screening were repeated within each outer training fold. Missing numerical predictors were replaced by the corresponding training-fold median and accompanied by a missingness indicator; missing categorical values were treated as a separate category. Penalised regression parameters were selected using five-fold cross-validation within the training data, whereas random forest and XGBoost hyperparameters were tuned using three-fold internal cross-validation. Candidate configurations were compared on identical outer folds, and one configuration was retained for each outcome after jointly considering mean outer-fold ROC AUC and Brier score.

Performance was calculated exclusively from out-of-fold predictions and described using the area under the receiver operating characteristic curve, area under the precision–recall curve, Brier score, log loss, calibration intercept and calibration slope. Decision-curve analysis was used to assess net benefit across clinically relevant probability thresholds. Stratified folds were used to preserve outcome prevalence, and no over- or undersampling was applied. Machine-learning analyses were restricted to outcomes with at least 50 episodes in each outcome category. Anhydrous uric acid was present in only 25 episodes, which would have left approximately five positive episodes in each outer test fold and fewer in the inner validation folds; it was therefore excluded from machine-learning analysis.

The urinary variables used in the primary association and clustering analyses were complete for all 256 episodes. Serum association analyses used available cases without imputation. Missing predictor values in the prediction analyses were handled only within the corresponding outer training folds, as described above.

For unsupervised clustering, the 14 urinary measurements were standardised and subjected to principal-component analysis. The smallest number of principal components explaining at least 80% of the total variance was retained, followed by partitioning around medoids, which was chosen to reduce sensitivity to outlying metabolic profiles. Solutions containing two to six clusters were compared using mean silhouette width and minimum cluster size. The three-cluster solution was retained as a descriptive balance of separation, cluster size, stability and clinical interpretability; its stability was assessed in 50 bootstrap samples using Jaccard similarity. Stone composition was compared only after cluster assignment and did not influence the selection of the number of clusters.

All statistical tests were two-sided. *p* < 0.05 was considered statistically significant, except for the prespecified *p* < 0.10 retention criterion used in backward variable selection.

## 3. Results

### 3.1. Cohort and Mineral Composition Profile

All 510 operations contributed to the composition profile. The cohort included 346 operations for renal stones, 83 for ureteric stones, five for bladder stones and 76 for stones at multiple anatomical sites. Flexible ureteroscopy, including RIRS, accounted for 482 operations (94.5%), followed by semirigid ureteroscopic lithotripsy in 22 (4.3%), cystoscopic lithotripsy in five (1.0%) and percutaneous nephrolithotomy in one (0.2%). COM, COD, carbonate apatite and anhydrous uric acid were identified in 378 (74.1%), 355 (69.6%), 206 (40.4%) and 59 (11.6%) operations, respectively, with extensive overlap among calcium-containing minerals (Table S1; Figure 1b,c).

The metabolic cohort comprised 256 patients whose 297 eligible operations were reconstructed into 256 episodes. Compared with the 213 excluded operations, the 297 included operations had higher stone attenuation, shorter operative times, more frequent lower-calyx involvement and stone multiplicity, and less frequent preoperative ureteric stenting (Table S2). COM, COD, carbonate apatite and anhydrous uric acid were present in 201 (78.5%), 187 (73.0%), 101 (39.5%) and 25 (9.8%) episodes, respectively; individual episodes could contain more than one mineral (Figure 1d,e). Ten of the 25 anhydrous uric acid-positive episodes met the definition of pure uric acid stones.

The median age of the metabolic cohort was 55.0 years, and 161 patients (62.9%) were men. Median BMI was 24.7 kg/m^2^, median eGFR was 100.5 mL/min/1.73 m^2^ and median CSD was 19.8 mm [11.5–32.9]. Low urine volume was present in 173 episodes (67.6%), hypocitraturia in 144 (56.2%), hypercalciuria in 126 (49.2%) and hyperoxaluria in 75 (29.3%) (Table S3; Figure 1f). Because each episode could contribute to more than one component-positive group, the distributions of most urinary measurements overlapped across stone components. The most prominent descriptive contrasts involved lower urine pH and more frequent low-pH status for anhydrous uric acid, and higher urine pH and more frequent hypocitraturia for carbonate apatite; most other contrasts were smaller (Figure 2a,b).

### 3.2. Urinary Metabolic Abnormalities and Mineral-Specific Associations

Univariable associations of the prespecified adjustment variables and urinary metabolic candidates with each stone component are presented in Table S4. After adjustment for age, sex, BMI and eGFR, urine pH showed the clearest component-specific associations, with opposite directions for carbonate apatite and anhydrous uric acid. Each standard-deviation increase in urine pH was associated with higher odds of carbonate apatite (OR 1.78, 95% confidence interval [CI] 1.33–2.38; *p* < 0.001) and lower odds of anhydrous uric acid (OR 0.26, 95% CI 0.11–0.58; *p* = 0.001). Urine pH was selected in 98.8% and 97.8% of bootstrap samples for these respective outcomes. Urinary uric acid excretion was not independently associated with anhydrous uric acid.

COM was associated with lower urine pH (OR 0.69, 95% CI 0.50–0.96; *p* = 0.029) and higher urinary citrate (OR 1.58, 95% CI 1.02–2.42; *p* = 0.038). By contrast, carbonate apatite was associated with lower urinary citrate (OR 0.57, 95% CI 0.40–0.81; *p* = 0.002). The opposite citrate coefficients for COM and carbonate apatite prompted the mutually exclusive CaOx–CaP phenotype analysis described in the following section.

COD was associated with lower urine volume (OR 0.57, 95% CI 0.40–0.80; *p* = 0.001). Urinary calcium (OR 1.56, 95% CI 1.00–2.44; *p* = 0.0503) and citrate (OR 1.51, 95% CI 1.00–2.28; *p* = 0.052) were also retained under the prespecified *p* < 0.10 retention criterion, although neither provided strong evidence of an independent association at the conventional *p* < 0.05 threshold. The urinary variables retained in the final multivariable models are summarised in Table 1, and the complete model estimates are provided in Table S5.

### 3.3. Citrate Patterns Across Mutually Exclusive CaOx–CaP Phenotypes

Higher urine citrate was associated with COM in the component model (OR 1.58, 95% CI 1.02–2.42; *p* = 0.038), whereas lower citrate was associated with carbonate apatite (OR 0.57, 95% CI 0.40–0.81; *p* = 0.002). To assess whether accompanying minerals explained the opposing citrate coefficients, citrate status was compared across mutually exclusive mineral phenotypes. Hypocitraturia was present in 58 of 128 CaOx-only episodes (45.3%), 51 of 84 mixed CaOx–CaP episodes (60.7%) and 17 of 19 CaP-without-CaOx episodes (89.5%). It was also present in 8 of 10 uric acid-related episodes without CaOx (Figure 2c). Continuous urinary citrate and pH distributions across the mutually exclusive mineral phenotypes are shown in Figure 2d.

Thus, the COM-negative comparison group contained a disproportionate number of CaP-rich, low-citrate stones. The positive COM coefficient may therefore have reflected a contrast between mineral mixtures rather than a lithogenic effect of citrate.

### 3.4. Secondary Mineral Phenotypes and Serum Associations

By the EAU mineral definition, 103 episodes (40.2%) contained an infection-associated mineral, predominantly carbonate apatite. Seventy-nine episodes (30.9%) also had preoperative culture, nitrite or pyuria evidence of infection (Figure S1).

Available sample sizes for individual serum measurements ranged from 202 to 256 episodes, corresponding to 0–54 missing observations (0–21.1%) (Table S6). In secondary serum analyses, total carbon dioxide was associated with COD after false-discovery-rate correction (OR per standard deviation 1.58; raw *p* = 0.003; adjusted *p* = 0.037), and triglycerides were associated with anhydrous uric acid (OR 1.84; raw *p* = 0.003; adjusted *p* = 0.048). The association between serum uric acid and anhydrous uric acid was significant before correction (OR 1.72; *p* = 0.011) but attenuated after adjustment for multiple comparisons (*p* = 0.079).

### 3.5. Discrimination and Internal Structure of the Urinary Metabolic Profile

Candidate algorithms, predictor representations and screening strategies showed broadly similar internal-validation performance, although their relative rankings differed across outcomes (Figure S2). One configuration was therefore retained for each outcome after jointly considering mean outer-fold ROC AUC and Brier score, rather than applying a single algorithm to all three outcomes. The selected models used a random forest with continuous predictors after *p* < 0.20 training-fold screening for COM, an unscreened elastic-net model (α = 0.50) for COD, and an elastic-net model (α = 0.75) with continuous predictors after *p* < 0.20 screening for carbonate apatite. For COM, age, urine pH and eGFR had the largest permutation-importance values. In the elastic-net models, the largest COD coefficients involved male sex, urine volume, citrate and calcium, whereas urine pH and citrate had the largest coefficients for carbonate apatite (Figure 3a–c). Global SHAP importance showed similar leading predictors for COM and carbonate apatite but ranked citrate and calcium most highly for COD (Figure S4).

Out-of-fold areas under the receiver operating characteristic curve were 0.697, 0.654 and 0.712 for COM, COD and carbonate apatite, respectively (Figure 3d–f; Table S7). The corresponding precision–recall areas under the curve were 0.870, 0.823 and 0.591 (Figure S3a–c). Brier scores were 0.146, 0.180 and 0.209. Calibration intercepts were 0.30, −0.32 and 0.03, and calibration slopes were 0.80, 1.30 and 1.00, respectively. Calibration was closest to ideal for carbonate apatite, whereas the COM and COD models showed greater deviation (Figure 3g–i; Table S7). Decision-curve analysis showed that net benefit varied across outcomes and threshold probabilities (Figure S3d–f).

A comparison of solutions from two to six clusters supported a three-cluster solution after considering silhouette width, bootstrap stability and cluster size (Figure S5a). The selected PCA–PAM solution separated the 256 episodes into three partially overlapping clusters (Figure S5b). Cluster 1 comprised 64 episodes and was characterised by generally high urinary solute excretion and lower urine pH; cluster 2 comprised 106 episodes and showed an intermediate metabolic profile; and cluster 3 comprised 86 episodes and was characterised by lower solute excretion and higher urine pH (Figure S5c). Their bootstrap Jaccard values were 0.708, 0.611 and 0.694, respectively. Stone-component distributions overlapped substantially across the three clusters, and none represented a composition-specific metabolic subgroup (Figure S5d).

## 4. Discussion

In this consecutive surgical cohort, staged procedures were reconstructed as treatment episodes so that the complete baseline stone burden could be matched to FTIR-defined minerals and preoperative 24 h urine profiles. The analyses included component-specific associations, mutually exclusive mineral phenotypes, prediction modelling and unsupervised clustering. Urine pH emerged as the most stable axis of mineral differentiation. The apparent positive association between urinary citrate and COM, by contrast, was driven largely by the lower citrate levels of CaP-rich stones in the reference group. Machine-learning and clustering analyses provided limited additional separation between mineral components.

The pH axis is consistent with established physicochemical principles. At low pH, a greater proportion of urinary urate is protonated to poorly soluble uric acid; at higher pH, calcium-phosphate supersaturation rises [6,10,11]. These relationships underlie urinary alkalinisation for uric acid dissolution and prevention, as well as the concern that excessive alkalinisation may favour CaP crystallisation. The same component-specific directions have been observed in clinical studies: CaP stone formers generally have higher urine pH, whereas uric acid stone formers have substantially lower values [10,12,18]. The present results extend these observations to an episode-based analysis in which staged procedures were consolidated and mixed minerals were retained. Under this design, urine pH remained the most reproducible urinary correlate of both carbonate apatite and anhydrous uric acid. Urinary uric acid excretion was less informative than pH, consistent with the direct effect of pH on uric acid solubility and the greater short-term variability of uric acid excretion with diet and renal handling [9,11].

Citrate is an important inhibitor of calcium-stone formation: it complexes urinary calcium, lowers free calcium activity and restrains crystal nucleation, growth and aggregation. Hypocitraturia therefore contributes to both CaOx and CaP stone risk, but it may also influence which calcium mineral predominates. Previous work found lower urine citrate and higher urine pH in CaP than in CaOx stone formers, with these two measurements providing the strongest urinary discrimination between the phenotypes [10]. The same gradient was observed across the mutually exclusive phenotypes in the present cohort. Hypocitraturia increased from 45.3% in CaOx-only episodes to 60.7% in mixed CaOx–CaP and 89.5% in CaP without CaOx, becoming progressively more prominent as mineral composition shifted towards CaP.

This gradient may explain the apparently paradoxical positive citrate coefficient in the COM model. The COM-negative reference group included many CaP-rich stones with lower citrate, consistent with previous comparisons of CaP and CaOx stone formers [10]; consequently, a relative comparison could yield a positive COM coefficient without implying that citrate promotes COM crystallisation. Retaining the accompanying minerals is therefore important when interpreting component-specific citrate associations.

COM and COD were less clearly separated. Lower urine volume was associated with COD, while urine calcium and citrate were borderline. COM and COD differ in crystal structure and may be favoured by partly different metabolic and crystallisation conditions, although both phases commonly coexist within the same calculus [19,20]. Clinical studies, however, have not identified a uniform metabolic distinction between them. In 96 patients assessed using two pretreatment 24 h urine collections, Asplin et al. found that stones containing COD were associated with higher urine calcium and lower urine citrate [14]. A more recent study of 98 calcium oxalate stone formers found no broad differences in 24 h urine chemistry between COM and COD groups apart from urinary oxalate, although an unsupervised analysis identified a COM-enriched cluster with higher excretion of several urinary solutes [13]. In a separate study of 777 patients, urea, calcium and oxalate were the leading predictors of COM versus COD, but the resulting machine-learning model achieved only modest agreement (κ = 0.204) [21]. The association between COD and higher urine calcium in the present cohort therefore agrees with some earlier findings, whereas the lower urine volume and borderline positive citrate coefficient have not been reproduced consistently. Differences in stone definitions—pure or predominant composition versus the presence of a mineral within a mixed stone—and in the timing and number of urine collections may partly explain this variation [13,19,20]. Because the present data recorded component presence rather than spatial distribution or abundance, coexistence of COM and COD may also have limited their separation by a single preoperative urine profile.

The infection-stone analysis illustrates another danger of equating a mineral label with a clinical diagnosis. EAU guidance groups carbonate apatite, struvite and ammonium urate as infection-associated minerals, yet carbonate apatite can also arise in a non-infectious alkaline CaP environment [7,8]. Of 103 episodes meeting the mineral definition, 79 had contemporaneous evidence of infection. The remaining episodes may represent non-infectious CaP formation or infection not captured at the time of testing, particularly after antibacterial exposure. Culture, nitrite and pyuria are not interchangeable measures, and their sensitivity depends on organism type, prior treatment and sampling time. Carbonate apatite should therefore raise suspicion of infection but should not be interpreted as evidence of active infection in isolation [22,23].

Several associations involving demographic characteristics and kidney function were also consistent with previous clinical observations. Male sex was positively associated with COM and COD but inversely associated with carbonate apatite, in keeping with the greater representation of CaP stones among women reported in other cohorts [10,18]. Lower eGFR was associated with anhydrous uric acid, and eGFR has likewise contributed to a clinical model distinguishing pure uric acid stones from other compositions [24]. Diabetes and insulin resistance have been linked particularly to uric acid lithogenesis, partly through impaired renal ammonium excretion and persistently acidic urine [11,25,26]. Declining kidney function also alters the urinary excretion of several lithogenic solutes [17]. These observations provide a broader metabolic context for the association between lower eGFR and anhydrous uric acid in the present cohort, although the small number of positive episodes limits component-specific inference.

The serum analyses were exploratory. Higher triglycerides were associated with anhydrous uric acid, but this estimate was based on only 25 positive episodes. The direction is compatible with the association of uric acid nephrolithiasis with insulin resistance and persistently acidic urine [25,26,27]. However, triglycerides are more appropriately interpreted as a marker of systemic metabolic dysfunction than as a direct mediator of crystallisation. Serum uric acid showed a similar direction but did not remain significant after FDR correction. The total carbon dioxide–COD association lacks crystal-phase-specific precedent and was not accompanied by a corresponding urinary acid–base signal. These serum findings require replication and should not be interpreted as evidence of new causal mechanisms.

The timing of urine sampling also affects interpretation. Because the urine measurements were obtained before stone removal, precipitation into an existing calculus may have reduced the measured excretion of selected lithogenic solutes. In a prospective paired study, urinary calcium and phosphate increased after complete stone removal, whereas the other measured urinary parameters showed no significant group-level change [28]. The associations observed here should therefore be interpreted as contemporaneous relationships between the preoperative urinary environment and the retrieved mineral components, rather than as a reconstruction of the entire period of stone growth.

Previous attempts to infer stone composition from readily measurable variables have followed both imaging-based and clinical approaches. Non-contrast CT attenuation combined with urine pH can distinguish predominantly uric acid stones from calcium stones, particularly when low attenuation and acidic urine occur together [12]. At this centre, dual-source dual-energy CT achieved 97.5% accuracy in identifying the major stone component [29]. Other investigators have combined demographic characteristics, comorbidities, serum measurements and 24-hour urine chemistry. In 1296 patients, models based on electronic health records and urine measurements distinguished calcium from non-calcium stones with an AUC of 0.80; uric acid supersaturation, calcium-phosphate supersaturation and urinary ammonium were among the most informative predictors [30]. An eight-variable model incorporating age, sex, gout, diabetes, BMI, eGFR, bacteriuria and urine pH also reported high discrimination for pure uric acid stones [24]. These results were obtained for comparatively broad, mutually exclusive outcomes—particularly pure uric acid stones or calcium versus non-calcium stones—and are not directly comparable with overlapping component-presence outcomes in a cohort dominated by mixed stones.

After nested tuning, AUCs remained between 0.65 and 0.71, lower than those achieved in the previous anatomical-location study [15]. Composition models have also shown variable performance elsewhere: only one of five stone-type models retained performance across internal and external validation in a multicentre study [31], and agreement for COM versus COD was low in the Chmiel model [21]. Overlapping component-presence outcomes in mixed stones, changes in urinary exposure during stone growth, and unmeasured variation in diet, bowel disease, urinary supersaturation and mineral abundance may have contributed to the limited discrimination in this cohort [1,6,8,32]. Clustering likewise followed total solute excretion and pH without defining composition-specific groups, whereas Gadzhiev et al. reported a COM-enriched high-excretion cluster [13]. Differences in cohort composition, mineral classification and the urinary variables measured may account for this discrepancy.

Using treatment episodes rather than individual operations prevented staged procedures from duplicating the same baseline stone burden and metabolic information. Retaining all identified minerals was particularly informative for mixed CaOx–CaP stones, in which the accompanying minerals altered the apparent citrate association.

Several limitations should be considered. Only 256 of the 443 patients in the consecutive surgical cohort had the complete preoperative urine evaluation required for the episode analysis, and patients selected for metabolic testing may have differed systematically from those without complete testing. The retrospective, single-centre surgical setting further limits generalisability to nonsurgical stone formers and other institutions. Preoperative urine measurements may not capture long-term variation in urinary exposure. The potential influence of short-term treatment could not be fully excluded because the timing of antibacterial therapy relative to urine collection was not uniformly documented. Routine FTIR reports identified mineral components without providing their continuous proportions. The available FTIR data therefore allowed mineral coexistence to be examined, but not the relationship between mineral abundance and the strength of metabolic associations. The small number of episodes containing anhydrous uric acid limited the precision and complexity of the corresponding analyses. Finally, the variable-selection and prediction results were derived and internally evaluated in the same cohort and require confirmation in an independent population.

Stone composition and preoperative urine testing provided complementary information. The findings support considering the complete mineral profile alongside urine measurements, but this study did not assess whether such combined interpretation improves treatment decisions or prevents recurrence. These potential clinical benefits require prospective evaluation.

## 5. Conclusions

In this episode-based surgical cohort, urine pH showed opposite associations with carbonate apatite and anhydrous uric acid. Hypocitraturia was concentrated in CaP-containing phenotypes, which may explain the apparently positive citrate association with COM when all COM-negative stones served as the reference group. Preserving mixed mineral composition and consolidating staged procedures helped clarify these component–metabolism associations. Routine clinical and metabolic variables provided only moderate discrimination and cannot replace direct stone analysis. Whether joint interpretation of stone composition and urine measurements improves treatment decisions or recurrence outcomes requires prospective evaluation.

## Figures and Tables

**Figure 2:**
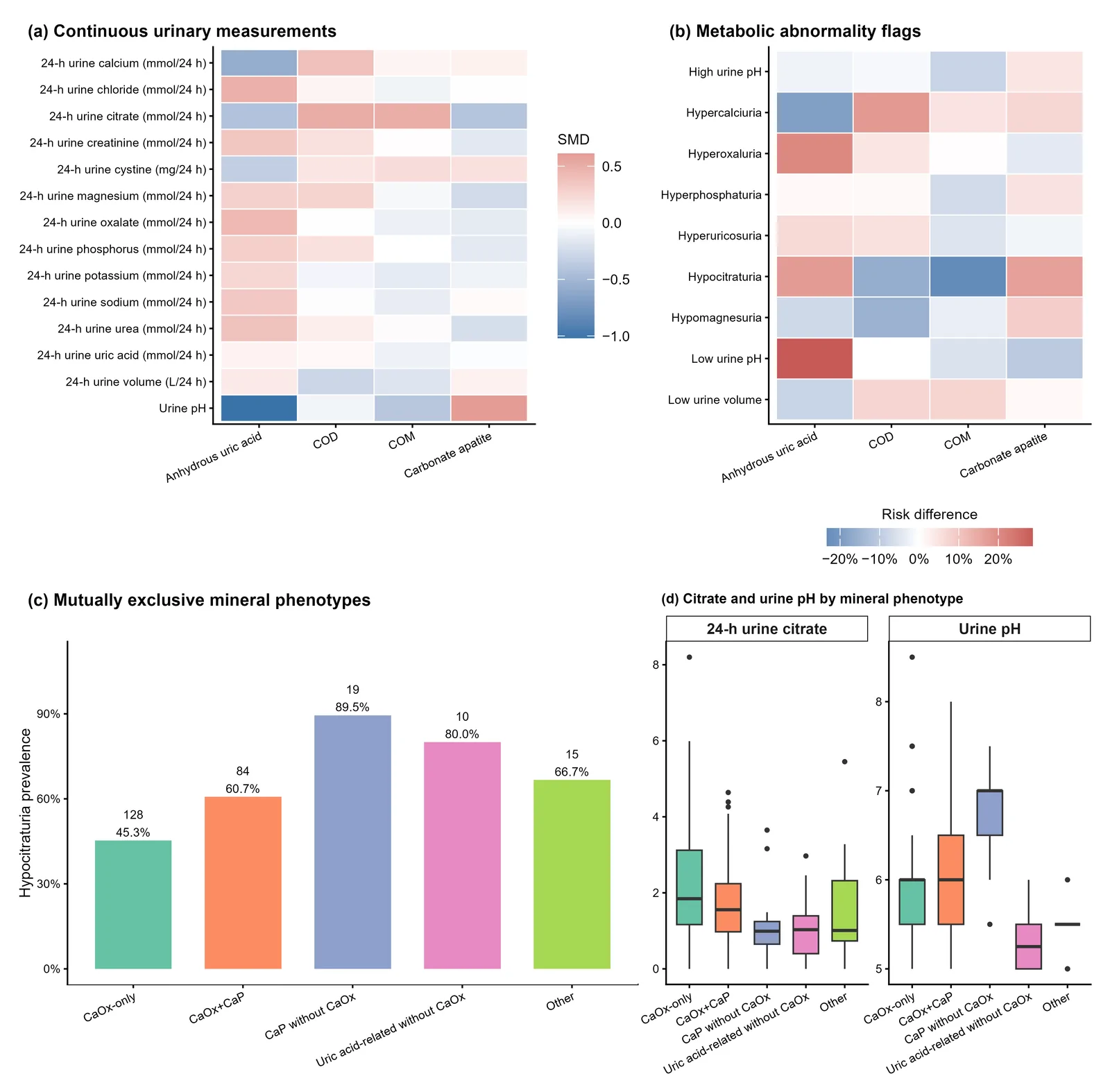
Component-specific urinary metabolic profiles and mutually exclusive mineral phenotypes. ((**a**) Standardised mean differences in continuous urinary measurements between component-positive and component-negative episodes; (**b**) absolute differences in the prevalence of urinary metabolic abnormalities between component-positive and component-negative episodes; (**c**) prevalence of hypocitraturia across mutually exclusive CaOx–CaP mineral phenotypes; (**d**) distributions of urinary citrate and urine pH across mutually exclusive mineral phenotypes).

**Figure 3:**
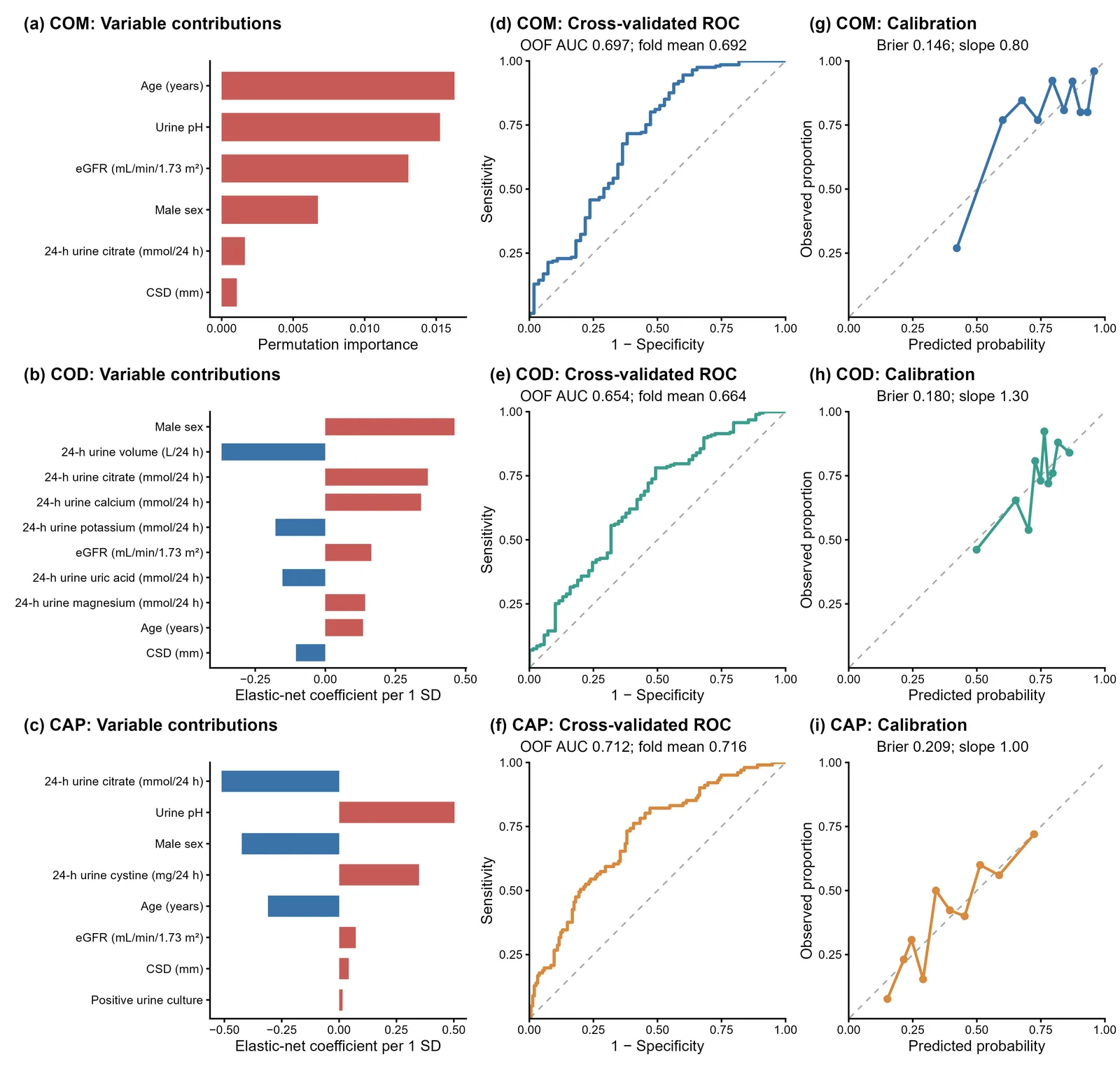
Model explanation, discrimination and calibration for predicting major stone components. ((**a**) Permutation importance of predictors in the random-forest model for COM; (**b**) standardised coefficients from the elastic-net model for COD; (**c**) standardised coefficients from the elastic-net model for carbonate apatite; (**d**–**f**) out-of-fold receiver operating characteristic curves for COM, COD and carbonate apatite, respectively; (**g**–**i**) out-of-fold calibration plots for COM, COD and carbonate apatite, respectively. Red and blue bars indicate positive and negative coefficients, respectively, in panels (**b**,**c**); all permutation-importance values in panel (**a**) are non-negative and are therefore shown in red. Blue, teal and orange curves and points represent COM, COD and carbonate apatite, respectively. Grey dashed lines indicate no discrimination in panels (**d**–**f**) and perfect calibration in panels (**g**–**i**). CAP, carbonate apatite).

**Table 1:** Component-specific associations of preoperative urinary metabolic characteristics with COM, COD, carbonate apatite and anhydrous uric acid.

Stone Component	Variable	Expression	Adjusted OR (95% CI)	*p* Value
COM	24 h urine citrate, mmol/24 h	Per 1 SD	1.58 (1.02–2.42)	0.038 *
COM	Urine pH	Per 1 SD	0.69 (0.50–0.96)	0.029 *
COD	24 h urine volume, L/24 h	Per 1 SD	0.57 (0.40–0.80)	0.001 *
COD	24 h urine calcium, mmol/24 h	Per 1 SD	1.56 (1.00–2.44)	0.0503
COD	24 h urine citrate, mmol/24 h	Per 1 SD	1.51 (1.00–2.28)	0.052
Carbonate apatite	Urine pH	Per 1 SD	1.78 (1.33–2.38)	<0.001 *
Carbonate apatite	24 h urine citrate, mmol/24 h	Per 1 SD	0.57 (0.40–0.81)	0.002 *
Anhydrous uric acid	Urine pH	Per 1 SD	0.26 (0.11–0.58)	0.001 *

* *p* < 0.05.

## Data Availability

The deidentified data supporting the findings of this study are available from the corresponding authors upon reasonable request and subject to institutional ethical approval. The data are not publicly available because of patient-privacy and ethical restrictions. The analysis code is available from the corresponding authors upon reasonable request.

## Supplementary Materials

The following supporting information can be downloaded at: https://www.mdpi.com/article/10.3390/metabo16100711/s1, Figure S1: Urinary metabolic abnormalities across derived stone phenotypes and infection-stone definitions; Figure S2: Comparison of candidate prediction strategies under nested cross-validation; Figure S3: Precision–recall performance and decision-curve analysis of the selected prediction models; Figure S4: SHAP-based interpretation of the selected prediction models; Figure S5: Robustness and characteristics of exploratory urinary metabolic clusters; Table S1: Complete clinical, stone and operative characteristics of 510 consecutive stone surgeries according to the four principal stone components; Table S2: Comparison of surgery records included in and excluded from the metabolic episode cohort; Table S3: Complete clinical, stone, operative and metabolic characteristics of 256 adjudicated metabolic-stone episodes according to the four principal stone components; Table S4: Univariable associations of prespecified adjustment variables and urinary candidates with the four principal stone components; Table S5: Complete coefficients from the final component-specific multivariable logistic regression models; Table S6: Available-case associations between serum metabolic markers and the four principal stone components; Table S7: Final prediction-model specifications and nested internal-validation performance.

## Abbreviations

The following abbreviations are used in this manuscript:

AUC	Area under the receiver operating characteristic curve
BMI	Body mass index
CaOx	Calcium oxalate
CaP	Calcium phosphate
CAP	Carbonate apatite
CI	Confidence interval
CKD-EPI	Chronic Kidney Disease Epidemiology Collaboration
COD	Calcium oxalate dihydrate
COM	Calcium oxalate monohydrate
CSD	Cumulative stone diameter
EAU	European Association of Urology
eGFR	Estimated glomerular filtration rate
FDR	False discovery rate
FTIR	Fourier-transform infrared spectroscopy
HPF	High-power field
IQR	Interquartile range
LASSO	Least absolute shrinkage and selection operator
OR	Odds ratio
PTH	Parathyroid hormone
WBC	White blood cell
XGBoost	Extreme gradient boosting
