# Supplementary material for: Linking Stone Mineral Composition to 24-Hour Urinary Metabolic Profiles: An Episode-Based Cohort Study
Source: Metabolites. 2026 Sep 24;16(10):711. doi: 10.3390/metabo16100711 (PMC13707276; doi:10.3390/metabo16100711)
Supplement: Supplementary file 1 [file metabolites-16-00711-s001.zip › metabolites-4536998-supplementary.pdf]

Supplementary Materials for
Linking Stone Mineral Composition to 24-Hour Urinary Metabolic Profiles: An Episode-Based Cohort Study

Shiwei Sun, Jiang Liu, Yi Qiao, Dong Wang, Lin Ma, Hongjun Li, and He Xiao

This file includes:

Supplementary Figures S1 to S5

Supplementary Tables S1 to S7

Supplementary Figures

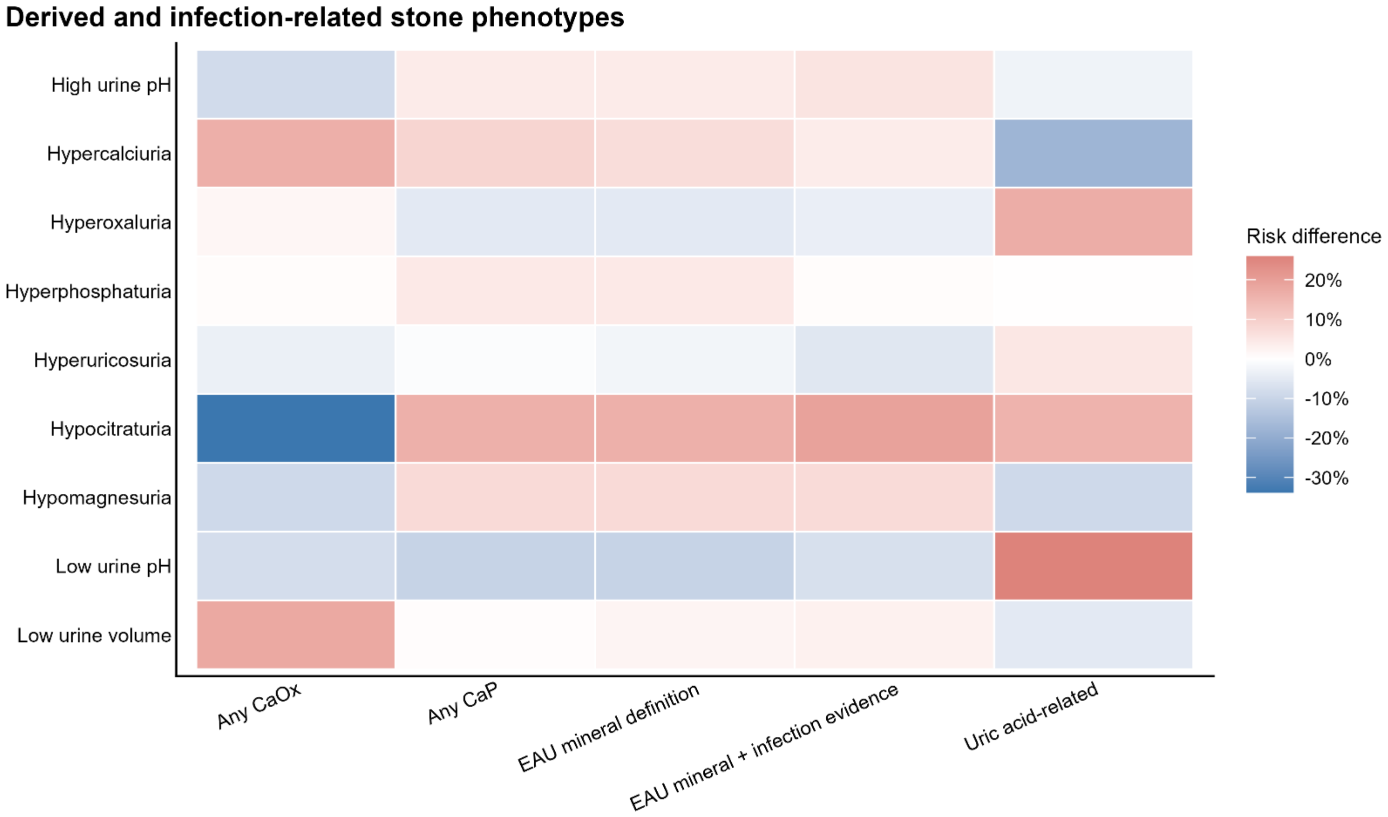

Figure S1. Urinary metabolic abnormalities across derived stone phenotypes and infection-stone definitions. (Absolute differences in the prevalence of nine urinary metabolic abnormalities for any CaOx, any CaP, the EAU infection-stone mineral definition, the EAU mineral definition accompanied by preoperative urinary infection evidence, and uric-acid-related stones.)

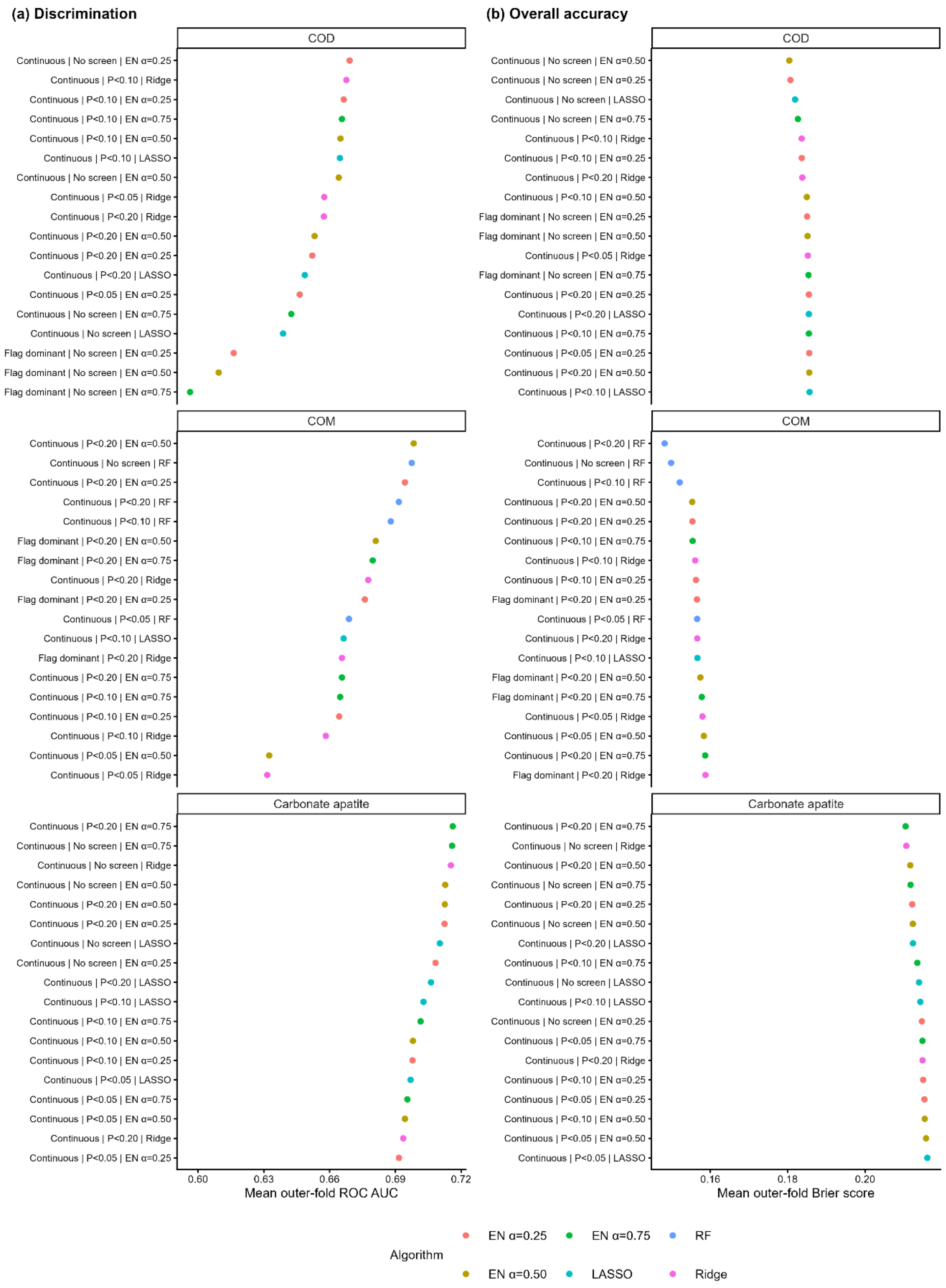

Figure S2. Comparison of candidate prediction strategies under nested cross-validation.

(a. Mean outer-fold receiver operating characteristic AUC across candidate algorithms, predictor representations and univariable screening strategies; b. Corresponding mean outer-fold Brier scores.)

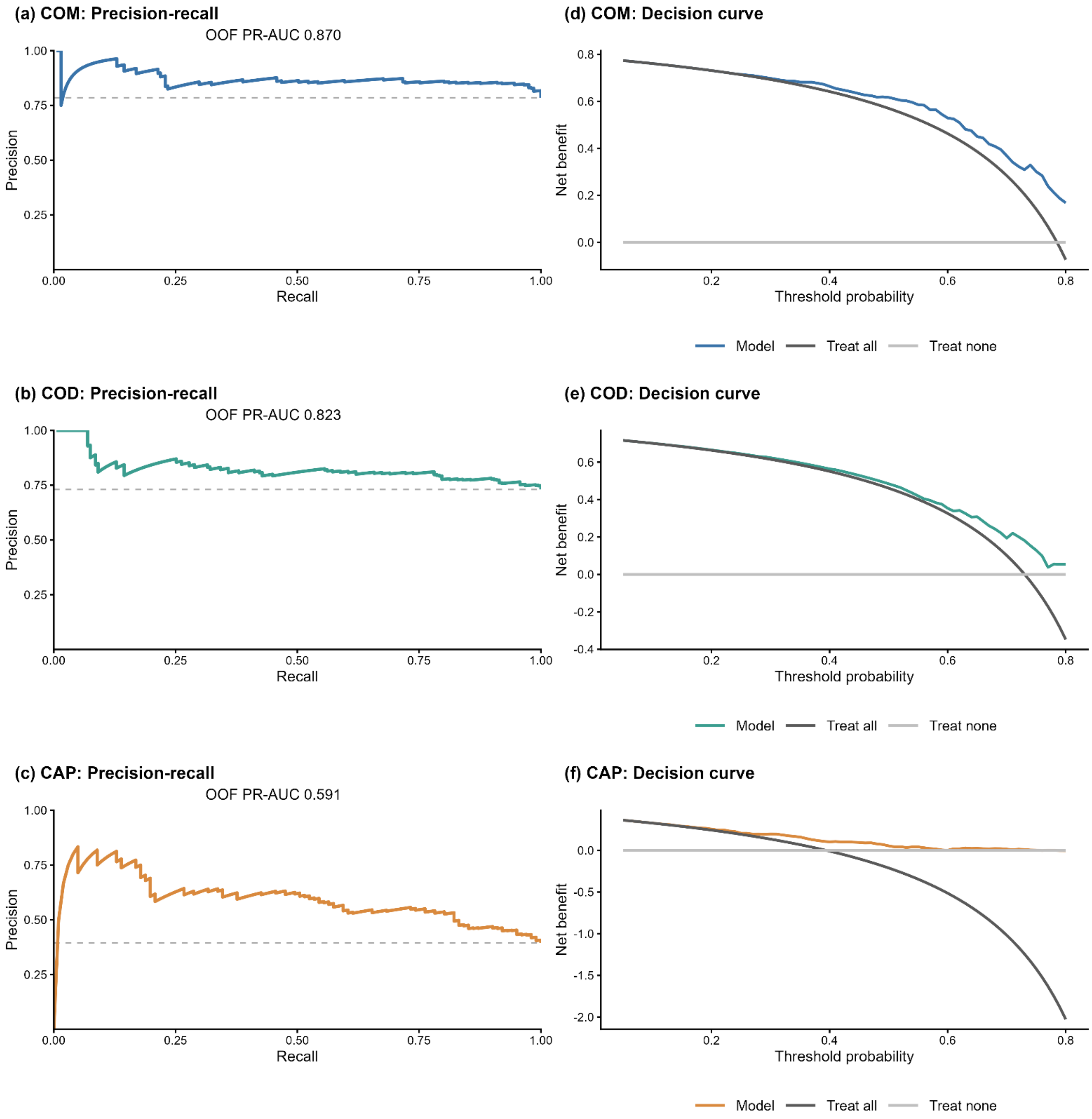

Figure S3. Precision–recall performance and decision-curve analysis of the selected prediction models.

(a–c. Out-of-fold precision–recall curves for COM, COD and carbonate apatite, respectively; d–f. Decision-curve analyses for COM, COD and carbonate apatite, respectively.)

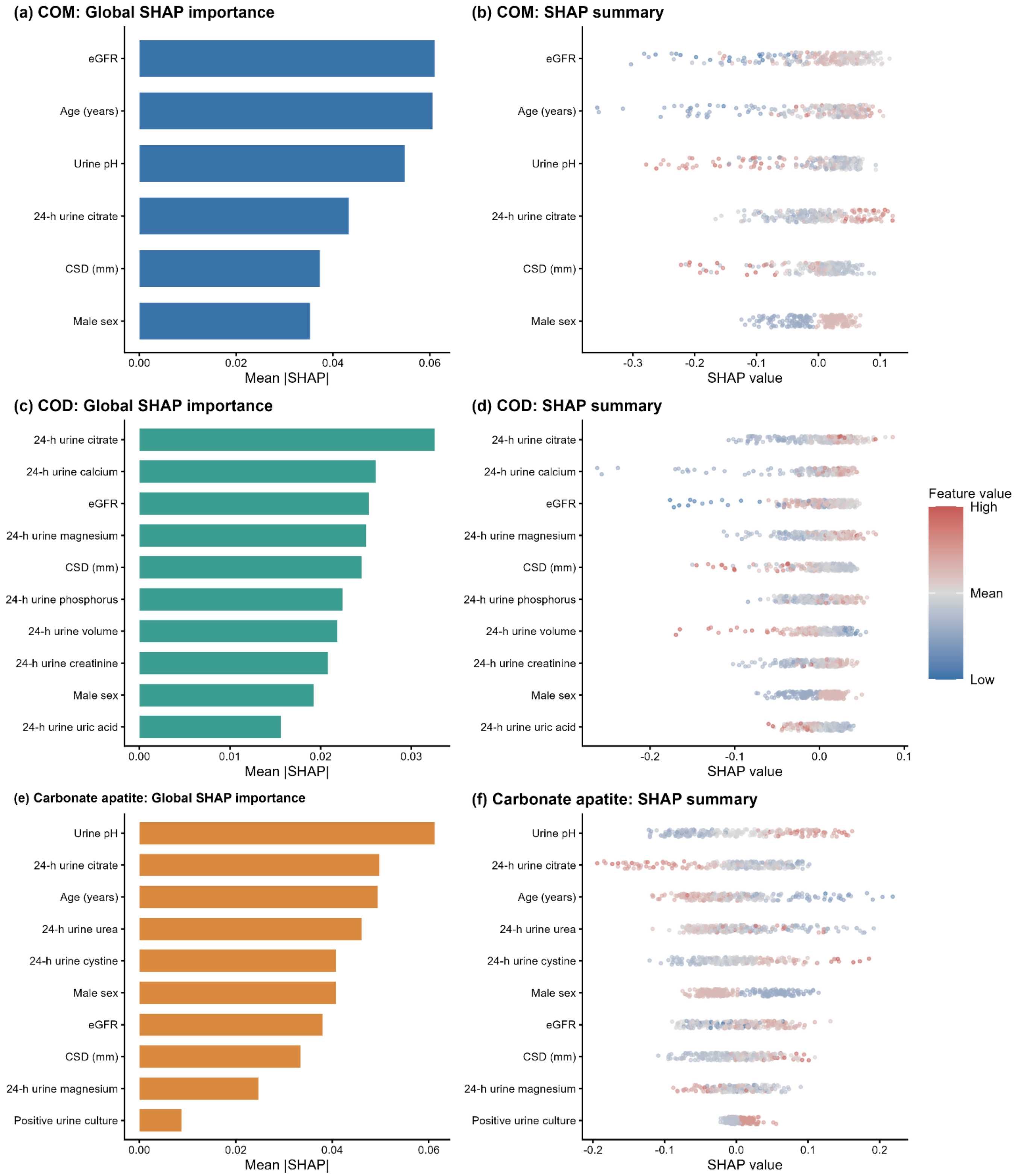

Figure S4. SHAP-based interpretation of the selected prediction models.

(a. Global SHAP importance for COM; b. SHAP summary plot for COM; c. Global SHAP importance for COD; d. SHAP summary plot for COD; e. Global SHAP importance for carbonate apatite; f. SHAP summary plot for carbonate apatite.) Global importance represents the mean absolute SHAP value.

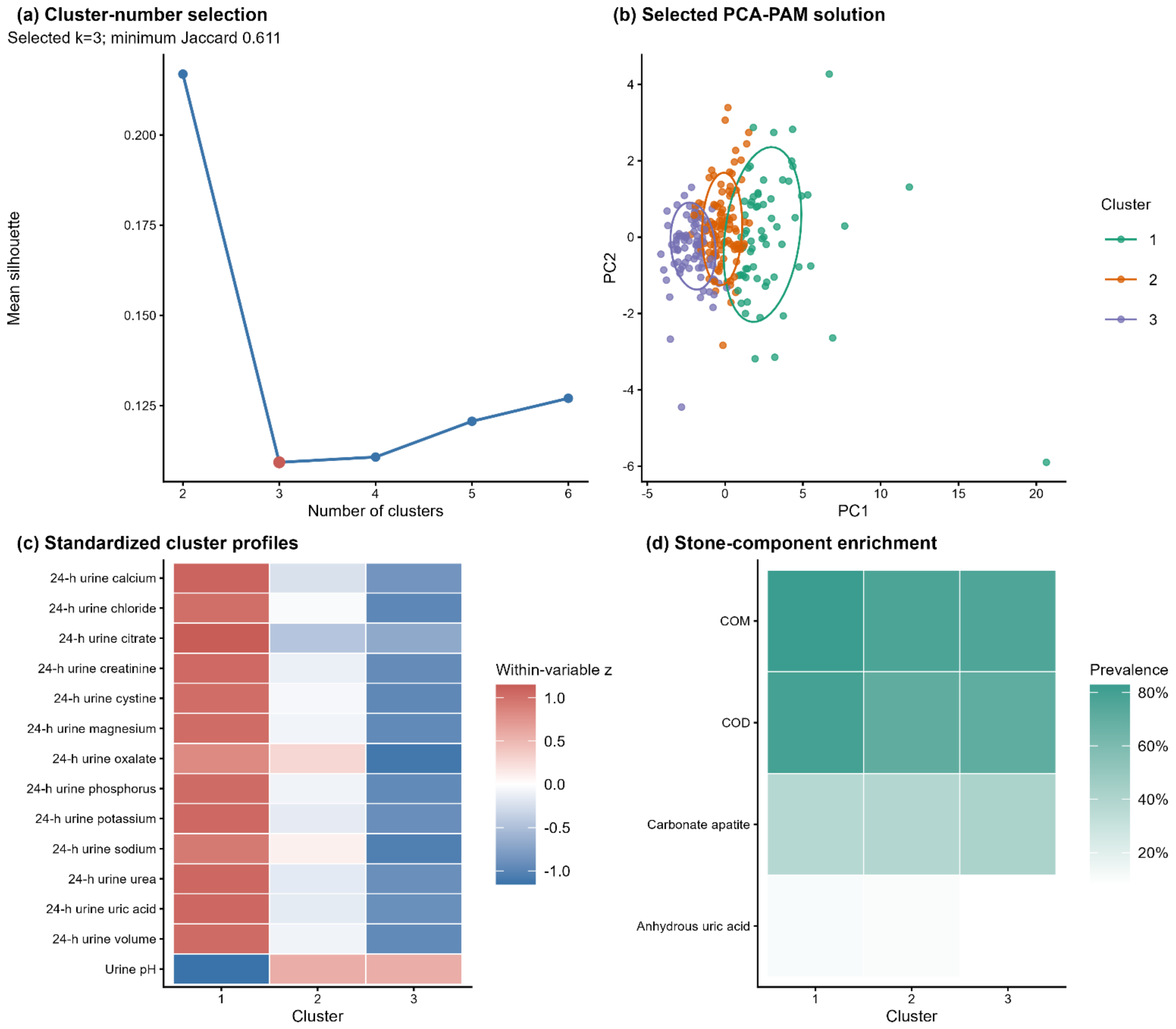

Figure S5. Robustness and characteristics of exploratory urinary metabolic clusters.

(a. Silhouette width for solutions with two to six clusters and bootstrap Jaccard stability of the selected three-cluster solution; b. Three-cluster partitioning-around-medoids solution projected onto the first two principal components, with 80% data ellipses; c. Standardised cluster-specific median profiles across 14 urinary measurements; d. Prevalence of COM, COD, carbonate apatite and anhydrous uric acid across the three clusters.)

Supplementary Tables

Table S1. Complete clinical, stone and operative characteristics of 510 consecutive stone surgeries according to the four principal stone components

Characteristic	Overall	COM Absent	COM Present	COM P value	COD Absent	COD Present	COD P value	Carbonate apatite Absent	Carbonate apatite Present	Carbonate apatite P value	Anhydrous uric acid Absent	Anhydrous uric acid Present	Anhydrous uric acid P value
**Demographic and clinical characteristics**													
Age, years	55.5 [46.0-64.0]	53.0 [40.0-64.2]	56.0 [46.2-63.8]	0.083	55.0 [44.0-65.0]	56.0 [46.0-63.0]	0.753	57.5 [49.0-65.0]	52.0 [41.0-62.0]	<0.001*	54.0 [45.0-63.0]	61.0 [55.0-66.5]	<0.001*
BMI, kg/m^2	25.1 [22.8-27.2]	25.6 [22.8-28.1]	24.9 [22.7-26.9]	0.113	25.0 [22.6-27.0]	25.2 [22.8-27.4]	0.746	25.2 [22.8-27.5]	24.9 [22.7-27.1]	0.464	24.8 [22.7-26.9]	26.4 [24.9-28.5]	<0.001*
eGFR, mL/min/1.73 m^2	99.2 [84.2-107.8]	98.0 [77.1-109.6]	99.8 [86.9-107.3]	0.272	95.7 [76.5-107.9]	100.3 [88.3-107.4]	0.011*	97.1 [82.5-105.1]	102.3 [90.5-112.2]	<0.001*	100.5 [88.3-108.6]	79.9 [60.9-95.0]	<0.001*
Sex, n (%)				<0.001*			<0.001*			<0.001*			0.032*
Female	176 (34.5%)	70 (53.0%)	106 (28.0%)		78 (50.3%)	98 (27.6%)		78 (25.7%)	98 (47.6%)		163 (36.1%)	13 (22.0%)	
Male	334 (65.5%)	62 (47.0%)	272 (72.0%)		77 (49.7%)	257 (72.4%)		226 (74.3%)	108 (52.4%)		288 (63.9%)	46 (78.0%)	
Hypertension, n (%)				0.847			0.924			0.162			0.011*
No	278 (54.5%)	71 (53.8%)	207 (54.8%)		84 (54.2%)	194 (54.6%)		158 (52.0%)	120 (58.3%)		255 (56.5%)	23 (39.0%)	
Yes	232 (45.5%)	61 (46.2%)	171 (45.2%)		71 (45.8%)	161 (45.4%)		146 (48.0%)	86 (41.7%)		196 (43.5%)	36 (61.0%)	
Diabetes mellitus, n (%)				0.036*			0.256			0.002*			0.0503
No	404 (79.2%)	113 (85.6%)	291 (77.0%)		118 (76.1%)	286 (80.6%)		227 (74.7%)	177 (85.9%)		363 (80.5%)	41 (69.5%)	
Yes	106 (20.8%)	19 (14.4%)	87 (23.0%)		37 (23.9%)	69 (19.4%)		77 (25.3%)	29 (14.1%)		88 (19.5%)	18 (30.5%)	
Coronary heart disease, n (%)				0.516			0.167			0.264			0.735
No	456 (89.4%)	120 (90.9%)	336 (88.9%)		143 (92.3%)	313 (88.2%)		268 (88.2%)	188 (91.3%)		404 (89.6%)	52 (88.1%)	
Yes	54 (10.6%)	12 (9.1%)	42 (11.1%)		12 (7.7%)	42 (11.8%)		36 (11.8%)	18 (8.7%)		47 (10.4%)	7 (11.9%)	
Hyperuricaemia or gout, n (%)				0.015*			0.300			0.456			0.009*
No	352 (69.0%)	80 (60.6%)	272 (72.0%)		102 (65.8%)	250 (70.4%)		206 (67.8%)	146 (70.9%)		320 (71.0%)	32 (54.2%)	
Yes	158 (31.0%)	52 (39.4%)	106 (28.0%)		53 (34.2%)	105 (29.6%)		98 (32.2%)	60 (29.1%)		131 (29.0%)	27 (45.8%)	
Renal tubular acidosis, n (%)				<0.001*			<0.001*			0.004*			0.524
No	504 (98.8%)	126 (95.5%)	378 (100.0%)		149 (96.1%)	355 (100.0%)		304 (100.0%)	200 (97.1%)		446 (98.9%)	58 (98.3%)	

Characteristic	Overall	COM Absent	COM Present	COM P value	COD Absent	COD Present	COD P value	Carbonate apatite Absent	Carbonate apatite Present	Carbonate apatite P value	Anhydrous uric acid Absent	Anhydrous uric acid Present	Anhydrous uric acid P value
Yes	6 (1.2%)	6 (4.5%)	0 (0.0%)		6 (3.9%)	0 (0.0%)		0 (0.0%)	6 (2.9%)		5 (1.1%)	1 (1.7%)	
Sjogren syndrome, n (%)				0.002*			<0.001*			0.168			1.000
No	501 (98.2%)	125 (94.7%)	376 (99.5%)		147 (94.8%)	354 (99.7%)		301 (99.0%)	200 (97.1%)		443 (98.2%)	58 (98.3%)	
Yes	9 (1.8%)	7 (5.3%)	2 (0.5%)		8 (5.2%)	1 (0.3%)		3 (1.0%)	6 (2.9%)		8 (1.8%)	1 (1.7%)	
Complex urological anatomy, n (%)				0.800			0.005*			0.574			0.691
No	441 (86.5%)	115 (87.1%)	326 (86.2%)		124 (80.0%)	317 (89.3%)		265 (87.2%)	176 (85.4%)		389 (86.3%)	52 (88.1%)	
Yes	69 (13.5%)	17 (12.9%)	52 (13.8%)		31 (20.0%)	38 (10.7%)		39 (12.8%)	30 (14.6%)		62 (13.7%)	7 (11.9%)	
**Stone and treatment characteristics**													
CSD, mm	20.0 [11.9-33.0]	24.9 [14.0-40.7]	17.6 [11.6-30.8]	0.003*	22.3 [11.9-40.0]	18.9 [11.9-31.1]	0.101	20.0 [11.7-33.3]	20.0 [12.5-32.7]	0.612	18.0 [11.7-32.0]	26.3 [19.5-46.2]	<0.001*
Stone attenuation, HU	1031.0 [935.0-1123.0]	993.0 [691.2-1063.2]	1040.0 [948.0-1146.0]	<0.001*	1034.0 [914.0-1164.0]	1030.5 [942.0-1104.0]	0.722	1023.0 [926.5-1100.5]	1040.0 [945.0-1135.8]	0.027*	1039.0 [946.2-1136.8]	599.0 [492.0-994.0]	<0.001*
Length of stay, days	7.0 [6.0-7.0]	7.0 [6.0-8.0]	7.0 [6.0-7.0]	0.016*	7.0 [6.0-8.0]	7.0 [6.0-7.0]	0.003*	7.0 [6.0-7.0]	7.0 [6.0-7.0]	0.225	7.0 [6.0-7.0]	7.0 [6.0-8.0]	0.450
Operative time, min	66.0 [42.0-99.0]	72.0 [42.8-125.2]	64.0 [42.0-94.8]	0.128	69.0 [45.0-106.0]	65.0 [42.0-97.0]	0.384	68.5 [42.8-105.2]	63.0 [42.0-93.0]	0.221	64.0 [42.0-92.0]	106.0 [55.0-153.5]	<0.001*
Stone location, n (%)				0.004*			0.401			0.898			0.075
Kidney	346 (67.8%)	92 (69.7%)	254 (67.2%)		103 (66.5%)	243 (68.5%)		205 (67.4%)	141 (68.4%)		302 (67.0%)	44 (74.6%)	
Ureter	83 (16.3%)	19 (14.4%)	64 (16.9%)		28 (18.1%)	55 (15.5%)		49 (16.1%)	34 (16.5%)		78 (17.3%)	5 (8.5%)	
Bladder	5 (1.0%)	5 (3.8%)	0 (0.0%)		3 (1.9%)	2 (0.6%)		4 (1.3%)	1 (0.5%)		3 (0.7%)	2 (3.4%)	
Multiple sites	76 (14.9%)	16 (12.1%)	60 (15.9%)		21 (13.5%)	55 (15.5%)		46 (15.1%)	30 (14.6%)		68 (15.1%)	8 (13.6%)	
Stone laterality, n (%)				0.002*			0.018*			0.214			0.135
Bilateral	50 (9.8%)	11 (8.3%)	39 (10.3%)		7 (4.5%)	43 (12.1%)		36 (11.8%)	14 (6.8%)		47 (10.4%)	3 (5.1%)	
Left	275 (53.9%)	63 (47.7%)	212 (56.1%)		86 (55.5%)	189 (53.2%)		159 (52.3%)	116 (56.3%)		244 (54.1%)	31 (52.5%)	
Not applicable	5 (1.0%)	5 (3.8%)	0 (0.0%)		3 (1.9%)	2 (0.6%)		4 (1.3%)	1 (0.5%)		3 (0.7%)	2 (3.4%)	
Right	180 (35.3%)	53 (40.2%)	127 (33.6%)		59 (38.1%)	121 (34.1%)		105 (34.5%)	75 (36.4%)		157 (34.8%)	23 (39.0%)	
Lower calyx involvement, n (%)				0.512			0.877			0.422			0.120

Characteristic	Overall	COM Absent	COM Present	COM P value	COD Absent	COD Present	COD P value	Carbonate apatite Absent	Carbonate apatite Present	Carbonate apatite P value	Anhydrous uric acid Absent	Anhydrous uric acid Present	Anhydrous uric acid P value
No	185 (36.3%)	51 (38.6%)	134 (35.4%)		57 (36.8%)	128 (36.1%)		106 (34.9%)	79 (38.3%)		169 (37.5%)	16 (27.1%)	
Yes	325 (63.7%)	81 (61.4%)	244 (64.6%)		98 (63.2%)	227 (63.9%)		198 (65.1%)	127 (61.7%)		282 (62.5%)	43 (72.9%)	
Staghorn stone, n (%)				0.021*			0.060			0.651			<0.001*
No	440 (86.3%)	106 (80.3%)	334 (88.4%)		127 (81.9%)	313 (88.2%)		264 (86.8%)	176 (85.4%)		398 (88.2%)	42 (71.2%)	
Yes	70 (13.7%)	26 (19.7%)	44 (11.6%)		28 (18.1%)	42 (11.8%)		40 (13.2%)	30 (14.6%)		53 (11.8%)	17 (28.8%)	
Multiple stones, n (%)				0.169			0.785			0.742			0.424
No	206 (40.4%)	60 (45.5%)	146 (38.6%)		64 (41.3%)	142 (40.0%)		121 (39.8%)	85 (41.3%)		185 (41.0%)	21 (35.6%)	
Yes	304 (59.6%)	72 (54.5%)	232 (61.4%)		91 (58.7%)	213 (60.0%)		183 (60.2%)	121 (58.7%)		266 (59.0%)	38 (64.4%)	
Preoperative D-J stent, n (%)				0.015*			0.008*			0.518			0.019*
No	345 (67.6%)	78 (59.1%)	267 (70.6%)		92 (59.4%)	253 (71.3%)		209 (68.8%)	136 (66.0%)		313 (69.4%)	32 (54.2%)	
Yes	165 (32.4%)	54 (40.9%)	111 (29.4%)		63 (40.6%)	102 (28.7%)		95 (31.2%)	70 (34.0%)		138 (30.6%)	27 (45.8%)	
Stone-free success, n (%)				0.456			0.244			0.928			0.747
No	172 (33.7%)	48 (36.4%)	124 (32.8%)		58 (37.4%)	114 (32.1%)		103 (33.9%)	69 (33.5%)		151 (33.5%)	21 (35.6%)	
Yes	338 (66.3%)	84 (63.6%)	254 (67.2%)		97 (62.6%)	241 (67.9%)		201 (66.1%)	137 (66.5%)		300 (66.5%)	38 (64.4%)	
**Preoperative urinary infection indicators**													
Preoperative pyuria, n (%)				0.081			0.305			0.742			0.577
No	154 (30.3%)	32 (24.2%)	122 (32.4%)		42 (27.1%)	112 (31.6%)		90 (29.7%)	64 (31.1%)		138 (30.7%)	16 (27.1%)	
Yes	355 (69.7%)	100 (75.8%)	255 (67.6%)		113 (72.9%)	242 (68.4%)		213 (70.3%)	142 (68.9%)		312 (69.3%)	43 (72.9%)	
Positive urine nitrite, n (%)				<0.001*			0.003*			0.392			0.786
No	473 (92.9%)	112 (84.8%)	361 (95.8%)		136 (87.7%)	337 (95.2%)		284 (93.7%)	189 (91.7%)		417 (92.7%)	56 (94.9%)	
Yes	36 (7.1%)	20 (15.2%)	16 (4.2%)		19 (12.3%)	17 (4.8%)		19 (6.3%)	17 (8.3%)		33 (7.3%)	3 (5.1%)	
Positive urine culture, n (%)				<0.001*			0.001*			0.104			0.923
No	369 (72.4%)	75 (56.8%)	294 (77.8%)		97 (62.6%)	272 (76.6%)		228 (75.0%)	141 (68.4%)		326 (72.3%)	43 (72.9%)	
Yes	141 (27.6%)	57 (43.2%)	84 (22.2%)		58 (37.4%)	83 (23.4%)		76 (25.0%)	65 (31.6%)		125 (27.7%)	16 (27.1%)	

* P<0.05.

Table S2. Comparison of surgery records included in and excluded from the metabolic episode cohort

Characteristic	Excluded	Included	P value	SMD
**Demographic and clinical characteristics**				
Age, years	55.0 [45.0-64.0]	56.0 [46.0-63.0]	0.546	0.10
BMI, kg/m^2	25.4 [23.1-27.4]	24.7 [22.7-27.2]	0.136	0.14
eGFR, mL/min/1.73 m^2	98.6 [84.6-108.2]	99.7 [84.2-107.4]	0.857	0.01
Sex, n (%)			0.778	0.03
Female	75 (35.2%)	101 (34.0%)		
Male	138 (64.8%)	196 (66.0%)		
Hypertension, n (%)			0.288	0.10
No	122 (57.3%)	156 (52.5%)		
Yes	91 (42.7%)	141 (47.5%)		
Diabetes mellitus, n (%)			0.108	0.15
No	176 (82.6%)	228 (76.8%)		
Yes	37 (17.4%)	69 (23.2%)		
Coronary heart disease, n (%)			0.650	0.04
No	192 (90.1%)	264 (88.9%)		
Yes	21 (9.9%)	33 (11.1%)		
Hyperuricaemia or gout, n (%)			0.696	0.04
No	145 (68.1%)	207 (69.7%)		
Yes	68 (31.9%)	90 (30.3%)		
Renal tubular acidosis, n (%)			0.043*	0.20
No	213 (100.0%)	291 (98.0%)		
Yes	0 (0.0%)	6 (2.0%)		
Sjogren syndrome, n (%)			0.087	0.18
No	212 (99.5%)	289 (97.3%)		
Yes	1 (0.5%)	8 (2.7%)		
Complex urological anatomy, n (%)			0.830	0.02
No	185 (86.9%)	256 (86.2%)		
Yes	28 (13.1%)	41 (13.8%)		
**Stone and treatment characteristics**				
CSD, mm	20.0 [12.4-34.0]	20.0 [11.7-32.3]	0.579	0.02
Stone attenuation, HU	1018.0 [926.2-1077.0]	1040.0 [941.5-1159.5]	0.019*	0.29
Length of stay, days	7.0 [6.0-7.0]	7.0 [6.0-7.0]	0.636	0.01
Operative time, min	74.0 [49.0-119.0]	61.0 [40.0-89.0]	<0.001*	0.39
Stone location, n (%)			0.063	0.17
Kidney	154 (72.3%)	192 (64.6%)		
Ureter	29 (13.6%)	54 (18.2%)		

Characteristic	Excluded	Included	P value	SMD
Bladder	4 (1.9%)	1 (0.3%)		
Multiple sites	26 (12.2%)	50 (16.8%)		
Stone laterality, n (%)			0.188	0.15
Bilateral	17 (8.0%)	33 (11.1%)		
Left	112 (52.6%)	163 (54.9%)		
Not applicable	4 (1.9%)	1 (0.3%)		
Right	80 (37.6%)	100 (33.7%)		
Lower calyx involvement, n (%)			0.017*	0.21
No	90 (42.3%)	95 (32.0%)		
Yes	123 (57.7%)	202 (68.0%)		
Staghorn stone, n (%)			0.326	0.09
No	180 (84.5%)	260 (87.5%)		
Yes	33 (15.5%)	37 (12.5%)		
Multiple stones, n (%)			0.001*	0.30
No	104 (48.8%)	102 (34.3%)		
Yes	109 (51.2%)	195 (65.7%)		
Preoperative D-J stent, n (%)			0.004*	0.26
No	129 (60.6%)	216 (72.7%)		
Yes	84 (39.4%)	81 (27.3%)		
Stone-free success, n (%)			0.466	0.07
No	68 (31.9%)	104 (35.0%)		
Yes	145 (68.1%)	193 (65.0%)		
**Preoperative urinary infection indicators**				
Preoperative pyuria, n (%)			0.716	0.03
No	66 (31.1%)	88 (29.6%)		
Yes	146 (68.9%)	209 (70.4%)		
Positive urine nitrite, n (%)			0.482	0.06
No	195 (92.0%)	278 (93.6%)		
Yes	17 (8.0%)	19 (6.4%)		
Positive urine culture, n (%)			0.823	0.02
No	153 (71.8%)	216 (72.7%)		
Yes	60 (28.2%)	81 (27.3%)		

* P<0.05.

Table S3. Complete clinical, stone, operative and metabolic characteristics of 256 adjudicated metabolic-stone episodes according to the four principal stone components

Characteristic	Overall	COM Absent	COM Present	COM P value	COD Absent	COD Present	COD P value	Carbonate apatite Absent	Carbonate apatite Present	Carbonate apatite P value	Anhydrous uric acid Absent	Anhydrous uric acid Present	Anhydrous uric acid P value
**Demographic and clinical characteristics**													
Age, years	55.0 [46.0-63.0]	51.0 [40.0-61.5]	57.0 [47.0-64.0]	0.009*	53.0 [44.0-63.0]	56.0 [47.0-63.5]	0.282	58.0 [49.0-65.0]	53.0 [42.0-61.0]	0.002*	55.0 [45.0-63.0]	60.0 [53.0-64.0]	0.150
BMI, kg/m^2	24.7 [22.6-27.3]	25.6 [22.6-28.1]	24.6 [22.6-27.1]	0.472	24.6 [22.2-26.7]	24.8 [22.7-27.5]	0.508	25.2 [22.9-27.5]	24.6 [22.2-27.2]	0.208	24.6 [22.6-27.1]	26.4 [24.5-28.7]	0.030*
eGFR, mL/min/1.73 m^2	100.5 [86.5-108.0]	100.3 [76.4-110.0]	100.5 [91.3-107.9]	0.539	100.2 [77.2-108.4]	100.6 [91.3-107.7]	0.225	98.3 [85.8-106.4]	102.7 [90.4-110.2]	0.039*	101.0 [91.4-108.3]	77.2 [54.7-99.3]	<0.001*
Sex, n (%)				<0.001*			<0.001*			<0.001*			0.153
Female	95 (37.1%)	31 (56.4%)	64 (31.8%)		37 (53.6%)	58 (31.0%)		44 (28.4%)	51 (50.5%)		89 (38.5%)	6 (24.0%)	
Male	161 (62.9%)	24 (43.6%)	137 (68.2%)		32 (46.4%)	129 (69.0%)		111 (71.6%)	50 (49.5%)		142 (61.5%)	19 (76.0%)	
Hypertension, n (%)				0.895			0.250			0.204			0.154
No	137 (53.5%)	29 (52.7%)	108 (53.7%)		41 (59.4%)	96 (51.3%)		78 (50.3%)	59 (58.4%)		127 (55.0%)	10 (40.0%)	
Yes	119 (46.5%)	26 (47.3%)	93 (46.3%)		28 (40.6%)	91 (48.7%)		77 (49.7%)	42 (41.6%)		104 (45.0%)	15 (60.0%)	
Diabetes mellitus, n (%)				0.037*			0.878			0.177			0.159
No	202 (78.9%)	49 (89.1%)	153 (76.1%)		54 (78.3%)	148 (79.1%)		118 (76.1%)	84 (83.2%)		185 (80.1%)	17 (68.0%)	
Yes	54 (21.1%)	6 (10.9%)	48 (23.9%)		15 (21.7%)	39 (20.9%)		37 (23.9%)	17 (16.8%)		46 (19.9%)	8 (32.0%)	
Coronary heart disease, n (%)				0.994			0.485			0.212			0.743
No	228 (89.1%)	49 (89.1%)	179 (89.1%)		63 (91.3%)	165 (88.2%)		135 (87.1%)	93 (92.1%)		206 (89.2%)	22 (88.0%)	
Yes	28 (10.9%)	6 (10.9%)	22 (10.9%)		6 (8.7%)	22 (11.8%)		20 (12.9%)	8 (7.9%)		25 (10.8%)	3 (12.0%)	
Hyperuricaemia or gout, n (%)				0.102			0.707			0.655			0.031*
No	181 (70.7%)	34 (61.8%)	147 (73.1%)		50 (72.5%)	131 (70.1%)		108 (69.7%)	73 (72.3%)		168 (72.7%)	13 (52.0%)	
Yes	75 (29.3%)	21 (38.2%)	54 (26.9%)		19 (27.5%)	56 (29.9%)		47 (30.3%)	28 (27.7%)		63 (27.3%)	12 (48.0%)	
Renal tubular acidosis, n (%)				<0.001*			0.001*			0.009*			0.404
No	251 (98.0%)	50 (90.9%)	201 (100.0%)		64 (92.8%)	187 (100.0%)		155 (100.0%)	96 (95.0%)		227 (98.3%)	24 (96.0%)	
Yes	5 (2.0%)	5 (9.1%)	0 (0.0%)		5 (7.2%)	0 (0.0%)		0 (0.0%)	5 (5.0%)		4 (1.7%)	1 (4.0%)	
Sjogren syndrome, n (%)				<0.001*			<0.001*			0.116			0.517
No	249 (97.3%)	49 (89.1%)	200 (99.5%)		62 (89.9%)	187 (100.0%)		153 (98.7%)	96 (95.0%)		225 (97.4%)	24 (96.0%)	
Yes	7 (2.7%)	6 (10.9%)	1 (0.5%)		7 (10.1%)	0 (0.0%)		2 (1.3%)	5 (5.0%)		6 (2.6%)	1 (4.0%)	
Complex urological anatomy, n (%)				0.321			0.011*			0.163			0.366

Characteristic	Overall	COM Absent	COM Present	COM P value	COD Absent	COD Present	COD P value	Carbonate apatite Absent	Carbonate apatite Present	Carbonate apatite P value	Anhydrous uric acid Absent	Anhydrous uric acid Present	Anhydrous uric acid P value
No	220 (85.9%)	45 (81.8%)	175 (87.1%)		53 (76.8%)	167 (89.3%)		137 (88.4%)	83 (82.2%)		200 (86.6%)	20 (80.0%)	
Yes	36 (14.1%)	10 (18.2%)	26 (12.9%)		16 (23.2%)	20 (10.7%)		18 (11.6%)	18 (17.8%)		31 (13.4%)	5 (20.0%)	
**Stone and treatment characteristics**													
CSD, mm	19.8 [11.5-32.9]	22.1 [12.5-38.8]	18.7 [11.4-32.2]	0.246	22.4 [12.0-40.6]	17.2 [11.3-32.0]	0.154	17.2 [10.0-31.6]	21.3 [12.9-35.1]	0.069	17.6 [11.1-32.0]	32.2 [17.0-48.8]	0.009*
Stone attenuation, HU	1040.5 [942.0-1166.2]	1037.5 [893.0-1167.5]	1042.5 [947.2-1164.0]	0.248	1052.5 [945.5-1221.8]	1033.5 [942.0-1139.0]	0.222	1026.5 [924.8-1116.5]	1053.5 [974.5-1192.8]	0.004*	1045.0 [947.0-1173.0]	665.0 [473.0-1052.0]	0.002*
Length of stay, days	7.0 [6.0-7.0]	7.0 [6.0-8.0]	7.0 [6.0-7.0]	0.617	7.0 [6.0-8.0]	7.0 [6.0-7.0]	0.275	7.0 [6.0-7.0]	7.0 [6.0-7.0]	0.725	7.0 [6.0-7.0]	7.0 [6.0-8.0]	0.459
Operative time, min	56.5 [38.0-81.2]	51.0 [35.0-79.0]	59.0 [38.0-83.0]	0.480	58.0 [40.0-79.0]	56.0 [37.5-83.5]	0.893	59.0 [37.0-84.0]	56.0 [39.0-79.0]	0.832	55.0 [37.5-78.0]	90.0 [44.0-143.0]	0.004*
Stone location, n (%)				0.427			0.410			0.845			0.392
Kidney	158 (61.7%)	33 (60.0%)	125 (62.2%)		42 (60.9%)	116 (62.0%)		97 (62.6%)	61 (60.4%)		140 (60.6%)	18 (72.0%)	
Ureter	50 (19.5%)	11 (20.0%)	39 (19.4%)		15 (21.7%)	35 (18.7%)		31 (20.0%)	19 (18.8%)		48 (20.8%)	2 (8.0%)	
Bladder	1 (0.4%)	1 (1.8%)	0 (0.0%)		1 (1.4%)	0 (0.0%)		1 (0.6%)	0 (0.0%)		1 (0.4%)	0 (0.0%)	
Multiple sites	47 (18.4%)	10 (18.2%)	37 (18.4%)		11 (15.9%)	36 (19.3%)		26 (16.8%)	21 (20.8%)		42 (18.2%)	5 (20.0%)	
Stone laterality, n (%)				0.342			0.048*			0.873			0.856
Bilateral	38 (14.8%)	8 (14.5%)	30 (14.9%)		5 (7.2%)	33 (17.6%)		25 (16.1%)	13 (12.9%)		34 (14.7%)	4 (16.0%)	
Left	132 (51.6%)	26 (47.3%)	106 (52.7%)		36 (52.2%)	96 (51.3%)		78 (50.3%)	54 (53.5%)		118 (51.1%)	14 (56.0%)	
Not applicable	1 (0.4%)	1 (1.8%)	0 (0.0%)		1 (1.4%)	0 (0.0%)		1 (0.6%)	0 (0.0%)		1 (0.4%)	0 (0.0%)	
Right	85 (33.2%)	20 (36.4%)	65 (32.3%)		27 (39.1%)	58 (31.0%)		51 (32.9%)	34 (33.7%)		78 (33.8%)	7 (28.0%)	
Lower calyx involvement, n (%)				0.726			0.934			0.940			0.111
No	88 (34.4%)	20 (36.4%)	68 (33.8%)		24 (34.8%)	64 (34.2%)		53 (34.2%)	35 (34.7%)		83 (35.9%)	5 (20.0%)	
Yes	168 (65.6%)	35 (63.6%)	133 (66.2%)		45 (65.2%)	123 (65.8%)		102 (65.8%)	66 (65.3%)		148 (64.1%)	20 (80.0%)	
Staghorn stone, n (%)				0.424			0.997			0.246			0.028*
No	230 (89.8%)	51 (92.7%)	179 (89.1%)		62 (89.9%)	168 (89.8%)		142 (91.6%)	88 (87.1%)		211 (91.3%)	19 (76.0%)	
Yes	26 (10.2%)	4 (7.3%)	22 (10.9%)		7 (10.1%)	19 (10.2%)		13 (8.4%)	13 (12.9%)		20 (8.7%)	6 (24.0%)	
Multiple stones, n (%)				0.861			0.457			0.190			0.204
No	91 (35.5%)	19 (34.5%)	72 (35.8%)		22 (31.9%)	69 (36.9%)		60 (38.7%)	31 (30.7%)		85 (36.8%)	6 (24.0%)	
Yes	165 (64.5%)	36 (65.5%)	129 (64.2%)		47 (68.1%)	118 (63.1%)		95 (61.3%)	70 (69.3%)		146 (63.2%)	19 (76.0%)	
Preoperative D-J stent, n (%)				0.067			0.022*			0.056			0.0504

Characteristic	Overall	COM Absent	COM Present	COM P value	COD Absent	COD Present	COD P value	Carbonate apatite Absent	Carbonate apatite Present	Carbonate apatite P value	Anhydrous uric acid Absent	Anhydrous uric acid Present	Anhydrous uric acid P value
No	212 (82.8%)	41 (74.5%)	171 (85.1%)		51 (73.9%)	161 (86.1%)		134 (86.5%)	78 (77.2%)		195 (84.4%)	17 (68.0%)	
Yes	44 (17.2%)	14 (25.5%)	30 (14.9%)		18 (26.1%)	26 (13.9%)		21 (13.5%)	23 (22.8%)		36 (15.6%)	8 (32.0%)	
Stone-free success, n (%)				0.811			0.128			0.225			0.228
No	85 (33.2%)	19 (34.5%)	66 (32.8%)		28 (40.6%)	57 (30.5%)		47 (30.3%)	38 (37.6%)		74 (32.0%)	11 (44.0%)	
Yes	171 (66.8%)	36 (65.5%)	135 (67.2%)		41 (59.4%)	130 (69.5%)		108 (69.7%)	63 (62.4%)		157 (68.0%)	14 (56.0%)	
**Preoperative urinary infection indicators**													
Preoperative pyuria, n (%)				0.393			0.739			0.358			0.365
No	82 (32.0%)	15 (27.3%)	67 (33.3%)		21 (30.4%)	61 (32.6%)		53 (34.2%)	29 (28.7%)		76 (32.9%)	6 (24.0%)	
Yes	174 (68.0%)	40 (72.7%)	134 (66.7%)		48 (69.6%)	126 (67.4%)		102 (65.8%)	72 (71.3%)		155 (67.1%)	19 (76.0%)	
Positive urine nitrite, n (%)				0.075			0.272			0.147			1.000
No	238 (93.0%)	48 (87.3%)	190 (94.5%)		62 (89.9%)	176 (94.1%)		147 (94.8%)	91 (90.1%)		214 (92.6%)	24 (96.0%)	
Yes	18 (7.0%)	7 (12.7%)	11 (5.5%)		7 (10.1%)	11 (5.9%)		8 (5.2%)	10 (9.9%)		17 (7.4%)	1 (4.0%)	
Positive urine culture, n (%)				0.232			0.150			0.112			0.156
No	184 (71.9%)	36 (65.5%)	148 (73.6%)		45 (65.2%)	139 (74.3%)		117 (75.5%)	67 (66.3%)		163 (70.6%)	21 (84.0%)	
Yes	72 (28.1%)	19 (34.5%)	53 (26.4%)		24 (34.8%)	48 (25.7%)		38 (24.5%)	34 (33.7%)		68 (29.4%)	4 (16.0%)	
**Episode-level treatment characteristics**													
Operations per episode	1.0 [1.0-1.0]	1.0 [1.0-1.0]	1.0 [1.0-1.0]	0.192	1.0 [1.0-1.0]	1.0 [1.0-1.0]	0.241	1.0 [1.0-1.0]	1.0 [1.0-1.0]	0.849	1.0 [1.0-1.0]	1.0 [1.0-2.0]	0.009*
Mixed stone composition, n (%)				<0.001*			<0.001*			0.325			<0.001*
No	29 (11.3%)	22 (40.0%)	7 (3.5%)		28 (40.6%)	1 (0.5%)		20 (12.9%)	9 (8.9%)		19 (8.2%)	10 (40.0%)	
Yes	227 (88.7%)	33 (60.0%)	194 (96.5%)		41 (59.4%)	186 (99.5%)		135 (87.1%)	92 (91.1%)		212 (91.8%)	15 (60.0%)	
Final stone-free success, n (%)				0.367			0.057			0.105			0.827
No	67 (26.2%)	17 (30.9%)	50 (24.9%)		24 (34.8%)	43 (23.0%)		35 (22.6%)	32 (31.7%)		60 (26.0%)	7 (28.0%)	
Yes	189 (73.8%)	38 (69.1%)	151 (75.1%)		45 (65.2%)	144 (77.0%)		120 (77.4%)	69 (68.3%)		171 (74.0%)	18 (72.0%)	
**Urinary metabolic measurements**													
24-h urine volume, L/24 h	2300.0 [1800.0-2800.0]	2300.0 [1850.0-3000.0]	2300.0 [1800.0-2700.0]	0.322	2300.0 [2000.0-3000.0]	2200.0 [1750.0-2750.0]	0.111	2200.0 [1750.0-2800.0]	2300.0 [1900.0-2800.0]	0.587	2300.0 [1800.0-2725.0]	2200.0 [1800.0-3000.0]	0.619
24-h urine calcium, mmol/24 h	5.0 [3.1-6.9]	4.8 [2.5-7.1]	5.0 [3.4-6.8]	0.642	4.1 [2.6-5.7]	5.1 [3.5-7.2]	0.005*	4.6 [2.8-6.7]	5.2 [3.7-7.0]	0.188	5.1 [3.4-7.0]	2.7 [1.8-5.2]	0.007*
24-h urine oxalate, mmol/24 h	0.4 [0.3-0.5]	0.4 [0.3-0.6]	0.4 [0.3-0.5]	0.700	0.4 [0.3-0.5]	0.4 [0.3-0.5]	0.882	0.4 [0.3-0.6]	0.4 [0.2-0.5]	0.129	0.4 [0.3-0.5]	0.5 [0.4-0.6]	0.026*
24-h urine citrate, mmol/24 h	1.6 [0.9-2.7]	1.3 [0.8-1.8]	1.7 [1.0-2.8]	0.004*	1.2 [0.7-2.0]	1.7 [1.1-2.8]	<0.001*	1.7 [1.0-3.0]	1.5 [0.9-2.0]	0.010*	1.6 [1.0-2.7]	1.0 [0.7-1.7]	0.015*

Characteristic	Overall	COM Absent	COM Present	COM P value	COD Absent	COD Present	COD P value	Carbonate apatite Absent	Carbonate apatite Present	Carbonate apatite P value	Anhydrous uric acid Absent	Anhydrous uric acid Present	Anhydrous uric acid P value
24-h urine uric acid, mmol/24 h	3.2 [2.4-4.0]	3.2 [2.5-4.0]	3.2 [2.4-4.0]	0.946	3.2 [2.4-3.9]	3.2 [2.5-4.1]	0.598	3.3 [2.4-4.1]	3.1 [2.4-4.0]	0.944	3.2 [2.5-4.0]	2.8 [2.2-4.1]	0.517
24-h urine magnesium, mmol/24 h	3.8 [2.9-4.7]	3.7 [2.7-4.9]	3.8 [2.9-4.7]	0.965	3.4 [2.7-4.3]	3.9 [3.1-4.9]	0.013*	3.9 [3.1-5.0]	3.6 [2.8-4.4]	0.082	3.7 [2.8-4.6]	4.3 [3.4-5.0]	0.165
24-h urine cystine, mg/24 h	71.0 [47.8-118.5]	68.0 [46.0-92.5]	72.0 [48.0-124.0]	0.337	69.0 [43.0-103.0]	72.0 [50.0-130.0]	0.102	70.0 [46.0-108.0]	72.0 [48.0-136.0]	0.239	72.0 [48.5-125.5]	61.0 [43.0-91.0]	0.132
24-h urine sodium, mmol/24 h	140.0 [110.0-188.2]	150.0 [118.0-190.5]	136.0 [108.0-185.0]	0.413	145.0 [118.0-189.0]	138.0 [106.0-187.5]	0.687	145.0 [106.0-186.0]	136.0 [115.0-189.0]	0.974	136.0 [108.0-183.5]	168.0 [129.0-225.0]	0.059
24-h urine potassium, mmol/24 h	40.4 [31.0-52.4]	42.4 [31.9-57.5]	39.2 [30.8-52.3]	0.343	39.1 [31.2-52.7]	41.0 [31.0-52.3]	0.791	40.5 [30.8-54.0]	40.3 [32.0-50.0]	0.920	39.4 [31.0-52.3]	42.4 [28.5-56.8]	0.420
24-h urine chloride, mmol/24 h	114.0 [87.0-158.0]	120.0 [93.5-169.0]	113.0 [87.0-158.0]	0.580	114.0 [95.0-150.0]	113.0 [86.5-158.5]	0.961	114.0 [86.5-158.0]	114.0 [90.0-156.0]	0.935	112.0 [86.5-155.0]	134.0 [121.0-192.0]	0.009*
24-h urine phosphorus, mmol/24 h	21.5 [16.1-27.8]	20.7 [15.9-27.2]	22.1 [16.2-27.8]	0.553	20.1 [15.9-24.3]	22.8 [16.1-28.6]	0.052	22.8 [17.1-28.0]	20.8 [15.4-26.6]	0.109	21.4 [15.9-27.6]	23.8 [19.9-30.4]	0.103
24-h urine urea, mmol/24 h	345.9 [274.4-436.6]	318.5 [274.4-431.4]	348.3 [275.0-438.0]	0.536	328.1 [271.8-430.5]	356.7 [275.9-441.8]	0.306	361.4 [285.6-445.1]	316.6 [256.2-418.5]	0.023*	344.0 [271.7-429.4]	378.8 [302.7-472.6]	0.066
24-h urine creatinine, mmol/24 h	11.6 [9.4-14.9]	11.5 [8.8-14.8]	11.6 [9.5-15.1]	0.720	10.7 [8.3-14.2]	12.1 [9.6-15.4]	0.061	12.1 [10.0-14.8]	10.8 [8.4-15.0]	0.071	11.5 [9.3-14.8]	14.1 [9.9-16.1]	0.077
Urine pH	6.0 [5.5-6.5]	6.0 [5.5-7.0]	6.0 [5.5-6.5]	0.024*	6.0 [5.5-6.5]	6.0 [5.5-6.5]	0.694	5.5 [5.5-6.0]	6.0 [5.5-6.5]	<0.001*	6.0 [5.5-6.5]	5.5 [5.0-5.5]	<0.001*
**Urinary metabolic abnormalities**													
Hypocitraturia, n (%)				0.002*			0.020*			0.009*			0.095
No	112 (43.8%)	14 (25.5%)	98 (48.8%)		22 (31.9%)	90 (48.1%)		78 (50.3%)	34 (33.7%)		105 (45.5%)	7 (28.0%)	
Yes	144 (56.2%)	41 (74.5%)	103 (51.2%)		47 (68.1%)	97 (51.9%)		77 (49.7%)	67 (66.3%)		126 (54.5%)	18 (72.0%)	
Hypercalciuria, n (%)				0.529			0.012*			0.273			0.070
No	130 (50.8%)	30 (54.5%)	100 (49.8%)		44 (63.8%)	86 (46.0%)		83 (53.5%)	47 (46.5%)		113 (48.9%)	17 (68.0%)	
Yes	126 (49.2%)	25 (45.5%)	101 (50.2%)		25 (36.2%)	101 (54.0%)		72 (46.5%)	54 (53.5%)		118 (51.1%)	8 (32.0%)	
Hyperoxaluria, n (%)				0.970			0.493			0.467			0.031*
No	181 (70.7%)	39 (70.9%)	142 (70.6%)		51 (73.9%)	130 (69.5%)		107 (69.0%)	74 (73.3%)		168 (72.7%)	13 (52.0%)	
Yes	75 (29.3%)	16 (29.1%)	59 (29.4%)		18 (26.1%)	57 (30.5%)		48 (31.0%)	27 (26.7%)		63 (27.3%)	12 (48.0%)	
Hypomagnesuria, n (%)				0.632			0.014*			0.134			0.434
No	188 (73.4%)	39 (70.9%)	149 (74.1%)		43 (62.3%)	145 (77.5%)		119 (76.8%)	69 (68.3%)		168 (72.7%)	20 (80.0%)	
Yes	68 (26.6%)	16 (29.1%)	52 (25.9%)		26 (37.7%)	42 (22.5%)		36 (23.2%)	32 (31.7%)		63 (27.3%)	5 (20.0%)	
Hyperuricosuria, n (%)				0.321			0.273			0.658			0.366
No	220 (85.9%)	45 (81.8%)	175 (87.1%)		62 (89.9%)	158 (84.5%)		132 (85.2%)	88 (87.1%)		200 (86.6%)	20 (80.0%)	

Characteristic	Overall	COM Absent	COM Present	COM P value	COD Absent	COD Present	COD P value	Carbonate apatite Absent	Carbonate apatite Present	Carbonate apatite P value	Anhydrous uric acid Absent	Anhydrous uric acid Present	Anhydrous uric acid P value
Yes	36 (14.1%)	10 (18.2%)	26 (12.9%)		7 (10.1%)	29 (15.5%)		23 (14.8%)	13 (12.9%)		31 (13.4%)	5 (20.0%)	
Hyperphosphaturia, n (%)				0.146			0.805			0.226			0.743
No	228 (89.1%)	46 (83.6%)	182 (90.5%)		62 (89.9%)	166 (88.8%)		141 (91.0%)	87 (86.1%)		206 (89.2%)	22 (88.0%)	
Yes	28 (10.9%)	9 (16.4%)	19 (9.5%)		7 (10.1%)	21 (11.2%)		14 (9.0%)	14 (13.9%)		25 (10.8%)	3 (12.0%)	
Low urine volume, n (%)				0.303			0.275			0.838			0.394
No	83 (32.4%)	21 (38.2%)	62 (30.8%)		26 (37.7%)	57 (30.5%)		51 (32.9%)	32 (31.7%)		73 (31.6%)	10 (40.0%)	
Yes	173 (67.6%)	34 (61.8%)	139 (69.2%)		43 (62.3%)	130 (69.5%)		104 (67.1%)	69 (68.3%)		158 (68.4%)	15 (60.0%)	
Low urine pH, n (%)				0.224			0.997			0.008*			<0.001*
No	230 (89.8%)	47 (85.5%)	183 (91.0%)		62 (89.9%)	168 (89.8%)		133 (85.8%)	97 (96.0%)		214 (92.6%)	16 (64.0%)	
Yes	26 (10.2%)	8 (14.5%)	18 (9.0%)		7 (10.1%)	19 (10.2%)		22 (14.2%)	4 (4.0%)		17 (7.4%)	9 (36.0%)	
High urine pH, n (%)				0.052			0.772			0.156			1.000
No	240 (93.8%)	48 (87.3%)	192 (95.5%)		64 (92.8%)	176 (94.1%)		148 (95.5%)	92 (91.1%)		216 (93.5%)	24 (96.0%)	
Yes	16 (6.2%)	7 (12.7%)	9 (4.5%)		5 (7.2%)	11 (5.9%)		7 (4.5%)	9 (8.9%)		15 (6.5%)	1 (4.0%)	
**Serum metabolic measurements**													
Serum calcium, mmol/L (n=244)	2.3 [2.2-2.3]	2.3 [2.2-2.3]	2.3 [2.2-2.3]	0.318	2.2 [2.2-2.3]	2.3 [2.2-2.3]	0.05003	2.3 [2.2-2.3]	2.3 [2.2-2.3]	0.736	2.3 [2.2-2.3]	2.2 [2.2-2.3]	0.183
Albumin-corrected calcium, mmol/L (n=244)	2.2 [2.2-2.3]	2.2 [2.2-2.3]	2.2 [2.2-2.3]	0.953	2.2 [2.2-2.3]	2.2 [2.2-2.3]	0.970	2.2 [2.2-2.3]	2.2 [2.2-2.3]	0.588	2.2 [2.2-2.3]	2.2 [2.2-2.3]	0.839
Serum phosphorus, mmol/L (n=239)	1.2 [1.0-1.3]	1.2 [1.1-1.3]	1.2 [1.0-1.3]	0.355	1.2 [1.1-1.3]	1.2 [1.0-1.2]	0.165	1.2 [1.0-1.2]	1.2 [1.1-1.3]	0.353	1.2 [1.0-1.3]	1.2 [1.1-1.2]	0.752
Serum magnesium, mmol/L (n=239)	0.9 [0.8-0.9]	0.8 [0.8-0.9]	0.9 [0.8-0.9]	0.787	0.9 [0.8-0.9]	0.9 [0.8-0.9]	0.832	0.9 [0.8-0.9]	0.9 [0.8-0.9]	0.819	0.9 [0.8-0.9]	0.8 [0.8-0.9]	0.990
Serum uric acid, µmol/L (n=241)	340.0 [294.0-398.0]	332.0 [290.0-417.0]	341.5 [296.8-394.0]	0.956	338.0 [285.0-389.0]	341.5 [297.0-402.8]	0.640	340.5 [300.5-410.8]	338.0 [287.5-382.0]	0.188	336.0 [291.0-390.0]	374.0 [314.8-451.8]	0.013*
Parathyroid hormone, pg/mL (n=236)	41.8 [31.4-54.3]	45.5 [31.0-53.8]	41.0 [31.4-54.6]	0.527	42.9 [28.9-57.2]	41.6 [32.5-53.2]	0.860	41.0 [31.5-53.0]	42.9 [31.4-57.2]	0.583	42.2 [31.5-53.9]	40.5 [31.0-67.1]	0.715
25-hydroxyvitamin D, ng/mL (n=202)	16.6 [12.5-22.4]	14.4 [11.6-22.6]	16.6 [12.6-22.3]	0.320	15.8 [12.4-24.2]	16.6 [12.5-22.2]	0.702	17.6 [13.1-22.6]	14.7 [11.6-21.9]	0.037*	16.1 [12.2-22.2]	18.6 [14.6-26.8]	0.111
Total carbon dioxide, mmol/L (n=243)	26.8 [25.1-28.3]	26.0 [24.8-28.3]	26.9 [25.3-28.3]	0.163	26.0 [24.7-27.7]	27.2 [25.4-28.5]	0.001*	27.0 [25.3-28.5]	26.2 [24.9-28.0]	0.079	26.9 [25.2-28.3]	25.9 [24.6-28.0]	0.225
Serum potassium, mmol/L (n=256)	3.9 [3.7-4.1]	3.8 [3.6-4.0]	3.9 [3.7-4.1]	0.049*	3.9 [3.7-4.1]	3.9 [3.7-4.1]	0.651	3.9 [3.8-4.1]	3.8 [3.7-4.0]	0.012*	3.9 [3.7-4.0]	4.0 [3.8-4.3]	0.158
Glucose, mmol/L (n=241)	5.0 [4.7-5.8]	4.9 [4.6-5.6]	5.1 [4.7-5.8]	0.091	4.9 [4.6-5.7]	5.1 [4.7-5.8]	0.110	5.1 [4.7-5.9]	4.9 [4.6-5.4]	0.051	5.0 [4.7-5.7]	5.4 [4.8-6.3]	0.136
Triglycerides, mmol/L (n=254)	1.3 [1.0-1.9]	1.4 [0.9-1.9]	1.3 [1.0-1.9]	0.890	1.1 [0.9-1.9]	1.4 [1.0-1.9]	0.349	1.3 [1.0-1.9]	1.3 [0.9-1.9]	0.558	1.3 [1.0-1.9]	1.4 [1.0-2.1]	0.337
Total cholesterol, mmol/L (n=254)	4.3 [3.5-5.0]	4.4 [3.7-5.1]	4.3 [3.5-4.9]	0.271	4.2 [3.5-5.0]	4.3 [3.5-4.9]	0.663	4.2 [3.4-4.9]	4.4 [3.8-5.0]	0.057	4.3 [3.5-5.0]	4.0 [3.4-4.8]	0.510

Characteristic	Overall	COM Absent	COM Present	COM P value	COD Absent	COD Present	COD P value	Carbonate apatite Absent	Carbonate apatite Present	Carbonate apatite P value	Anhydrous uric acid Absent	Anhydrous uric acid Present	Anhydrous uric acid P value
HDL cholesterol, mmol/L (n=254)	1.1 [0.9-1.3]	1.1 [1.0-1.4]	1.1 [0.9-1.3]	0.249	1.1 [1.0-1.2]	1.1 [0.9-1.3]	0.691	1.1 [0.9-1.3]	1.1 [0.9-1.3]	0.117	1.1 [0.9-1.3]	0.9 [0.8-1.1]	0.012*
LDL cholesterol, mmol/L (n=254)	2.4 [1.9-3.0]	2.4 [2.1-3.0]	2.4 [1.9-3.0]	0.624	2.5 [2.0-2.9]	2.4 [1.9-3.0]	0.791	2.4 [1.8-3.0]	2.6 [2.2-3.1]	0.063	2.5 [1.9-3.0]	2.2 [1.8-2.5]	0.020*

* $P<0.05$.

Table S4. Univariable associations of prespecified adjustment variables and urinary candidates with the four principal stone components

Stone component	Variable	Role	Expression	N/events	Unadjusted OR (95% CI)	P value
COM	Age, years	Prespecified adjustment variable	Per 1 SD	256/201	1.51 (1.10-2.06)	0.010*
COM	Male sex	Prespecified adjustment variable	Male vs female	256/201	2.76 (1.50-5.09)	0.001*
COM	BMI, kg/m^2	Prespecified adjustment variable	Per 1 SD	256/201	0.94 (0.70-1.27)	0.688
COM	eGFR, mL/min/1.73 m^2	Prespecified adjustment variable	Per 1 SD	256/201	1.26 (0.95-1.67)	0.111
COM	24-h urine volume, L/24 h	Urinary candidate variable	Per 1 SD	256/201	0.83 (0.62-1.11)	0.206
COM	24-h urine calcium, mmol/24 h	Urinary candidate variable	Per 1 SD	256/201	1.07 (0.78-1.46)	0.680
COM	24-h urine oxalate, mmol/24 h	Urinary candidate variable	Per 1 SD	256/201	0.90 (0.67-1.21)	0.474
COM	24-h urine citrate, mmol/24 h	Urinary candidate variable	Per 1 SD	256/201	1.75 (1.20-2.55)	0.004*
COM	Urine pH	Urinary candidate variable	Per 1 SD	256/201	0.67 (0.50-0.90)	0.007*
COD	Age, years	Prespecified adjustment variable	Per 1 SD	256/187	1.20 (0.91-1.59)	0.192
COD	Male sex	Prespecified adjustment variable	Male vs female	256/187	2.57 (1.46-4.53)	0.001*
COD	BMI, kg/m^2	Prespecified adjustment variable	Per 1 SD	256/187	1.15 (0.87-1.53)	0.323
COD	eGFR, mL/min/1.73 m^2	Prespecified adjustment variable	Per 1 SD	256/187	1.41 (1.08-1.84)	0.011*
COD	24-h urine volume, L/24 h	Urinary candidate variable	Per 1 SD	256/187	0.75 (0.57-0.99)	0.043*
COD	24-h urine calcium, mmol/24 h	Urinary candidate variable	Per 1 SD	256/187	1.61 (1.14-2.29)	0.007*
COD	24-h urine oxalate, mmol/24 h	Urinary candidate variable	Per 1 SD	256/187	0.99 (0.75-1.30)	0.929
COD	24-h urine citrate, mmol/24 h	Urinary candidate variable	Per 1 SD	256/187	1.80 (1.27-2.54)	<0.001*
COD	Urine pH	Urinary candidate variable	Per 1 SD	256/187	0.93 (0.71-1.22)	0.611
Carbonate apatite	Age, years	Prespecified adjustment variable	Per 1 SD	256/101	0.66 (0.50-0.85)	0.002*
Carbonate apatite	Male sex	Prespecified adjustment variable	Male vs female	256/101	0.39 (0.23-0.66)	<0.001*
Carbonate apatite	BMI, kg/m^2	Prespecified adjustment variable	Per 1 SD	256/101	0.86 (0.66-1.11)	0.237
Carbonate apatite	eGFR, mL/min/1.73 m^2	Prespecified adjustment variable	Per 1 SD	256/101	1.22 (0.94-1.59)	0.138
Carbonate apatite	Urine pH	Urinary candidate variable	Per 1 SD	256/101	1.86 (1.41-2.45)	<0.001*
Carbonate apatite	24-h urine citrate, mmol/24 h	Urinary candidate variable	Per 1 SD	256/101	0.65 (0.49-0.86)	0.003*
Carbonate apatite	24-h urine calcium, mmol/24 h	Urinary candidate variable	Per 1 SD	256/101	1.09 (0.85-1.40)	0.507
Carbonate apatite	24-h urine phosphorus, mmol/24 h	Urinary candidate variable	Per 1 SD	256/101	0.85 (0.65-1.12)	0.247
Anhydrous uric acid	Age, years	Prespecified adjustment variable	Per 1 SD	256/25	1.41 (0.93-2.15)	0.108
Anhydrous uric acid	Male sex	Prespecified adjustment variable	Male vs female	256/25	1.98 (0.76-5.16)	0.160
Anhydrous uric acid	BMI, kg/m^2	Prespecified adjustment variable	Per 1 SD	256/25	1.46 (0.98-2.17)	0.065
Anhydrous uric acid	eGFR, mL/min/1.73 m^2	Prespecified adjustment variable	Per 1 SD	256/25	0.41 (0.29-0.59)	<0.001*
Anhydrous uric acid	Urine pH	Urinary candidate variable	Per 1 SD	256/25	0.22 (0.11-0.46)	<0.001*
Anhydrous uric acid	24-h urine uric acid, mmol/24 h	Urinary candidate variable	Per 1 SD	256/25	1.08 (0.73-1.59)	0.701
Anhydrous uric acid	24-h urine volume, L/24 h	Urinary candidate variable	Per 1 SD	256/25	1.13 (0.76-1.70)	0.545

* P<0.05.

Table S5. Complete coefficients from the final component-specific multivariable logistic regression models

Stone component	Variable	Role	Expression	N/events	Adjusted OR (95% CI)	P value
COM	Age, years	Prespecified adjustment variable	Per 1 SD	256/201	1.75 (1.19-2.58)	0.005*
COM	Male sex	Prespecified adjustment variable	Male vs female	256/201	2.96 (1.50-5.85)	0.002*
COM	BMI, kg/m^2	Prespecified adjustment variable	Per 1 SD	256/201	0.80 (0.58-1.11)	0.187
COM	eGFR, mL/min/1.73 m^2	Prespecified adjustment variable	Per 1 SD	256/201	1.47 (1.01-2.12)	0.042*
COM	24-h urine citrate, mmol/24 h	Selected urinary variable	Per 1 SD	256/201	1.58 (1.02-2.42)	0.038*
COM	Urine pH	Selected urinary variable	Per 1 SD	256/201	0.69 (0.50-0.96)	0.029*
COD	Age, years	Prespecified adjustment variable	Per 1 SD	256/187	1.41 (0.98-2.04)	0.063
COD	Male sex	Prespecified adjustment variable	Male vs female	256/187	3.16 (1.63-6.11)	<0.001*
COD	BMI, kg/m^2	Prespecified adjustment variable	Per 1 SD	256/187	1.06 (0.77-1.45)	0.726
COD	eGFR, mL/min/1.73 m^2	Prespecified adjustment variable	Per 1 SD	256/187	1.33 (0.92-1.91)	0.132
COD	24-h urine volume, L/24 h	Selected urinary variable	Per 1 SD	256/187	0.57 (0.40-0.80)	0.001*
COD	24-h urine calcium, mmol/24 h	Selected urinary variable	Per 1 SD	256/187	1.56 (1.00-2.44)	0.0503
COD	24-h urine citrate, mmol/24 h	Selected urinary variable	Per 1 SD	256/187	1.51 (1.00-2.28)	0.052
Carbonate apatite	Age, years	Prespecified adjustment variable	Per 1 SD	256/101	0.73 (0.52-1.02)	0.063
Carbonate apatite	Male sex	Prespecified adjustment variable	Male vs female	256/101	0.38 (0.21-0.69)	0.002*
Carbonate apatite	BMI, kg/m^2	Prespecified adjustment variable	Per 1 SD	256/101	1.04 (0.77-1.39)	0.817
Carbonate apatite	eGFR, mL/min/1.73 m^2	Prespecified adjustment variable	Per 1 SD	256/101	1.22 (0.86-1.74)	0.266
Carbonate apatite	Urine pH	Selected urinary variable	Per 1 SD	256/101	1.78 (1.33-2.38)	<0.001*
Carbonate apatite	24-h urine citrate, mmol/24 h	Selected urinary variable	Per 1 SD	256/101	0.57 (0.40-0.81)	0.002*
Anhydrous uric acid	Age, years	Prespecified adjustment variable	Per 1 SD	256/25	0.75 (0.39-1.42)	0.375
Anhydrous uric acid	Male sex	Prespecified adjustment variable	Male vs female	256/25	2.23 (0.69-7.22)	0.180
Anhydrous uric acid	BMI, kg/m^2	Prespecified adjustment variable	Per 1 SD	256/25	1.19 (0.75-1.89)	0.464
Anhydrous uric acid	eGFR, mL/min/1.73 m^2	Prespecified adjustment variable	Per 1 SD	256/25	0.37 (0.23-0.59)	<0.001*
Anhydrous uric acid	Urine pH	Selected urinary variable	Per 1 SD	256/25	0.26 (0.11-0.58)	0.001*

* P<0.05.

Table S6. Available-case associations between serum metabolic markers and the four principal stone components

Stone component	Serum marker	N/events	OR per 1 SD (95% CI)	P value	FDR-adjusted P
COM	Serum calcium, mmol/L	244/191	1.16 (0.85-1.63)	0.370	0.610
COM	Albumin-corrected calcium, mmol/L	244/191	1.01 (0.75-1.40)	0.955	0.955
COM	Serum phosphorus, mmol/L	239/186	0.88 (0.65-1.20)	0.427	0.610
COM	Serum magnesium, mmol/L	239/186	1.09 (0.80-1.48)	0.582	0.741
COM	Serum uric acid, μmol/L	241/188	0.98 (0.72-1.34)	0.899	0.955
COM	Parathyroid hormone, pg/mL	236/186	0.85 (0.64-1.14)	0.246	0.610
COM	25-hydroxyvitamin D, ng/mL	202/159	1.18 (0.84-1.72)	0.366	0.610
COM	Total carbon dioxide, mmol/L	243/190	1.15 (0.85-1.57)	0.362	0.610
COM	Serum potassium, mmol/L	256/201	1.43 (1.05-1.99)	0.028	0.396
COM	Glucose, mmol/L	241/189	1.34 (0.95-2.00)	0.122	0.610
COM	Triglycerides, mmol/L	254/199	0.90 (0.68-1.21)	0.436	0.610
COM	Total cholesterol, mmol/L	254/199	0.87 (0.65-1.17)	0.352	0.610
COM	HDL cholesterol, mmol/L	254/199	0.84 (0.63-1.12)	0.227	0.610
COM	LDL cholesterol, mmol/L	254/199	0.95 (0.71-1.28)	0.709	0.827
COD	Serum calcium, mmol/L	244/179	1.46 (1.06-2.05)	0.025	0.177
COD	Albumin-corrected calcium, mmol/L	244/179	1.11 (0.83-1.53)	0.499	0.994
COD	Serum phosphorus, mmol/L	239/174	0.79 (0.59-1.06)	0.117	0.431
COD	Serum magnesium, mmol/L	239/176	0.96 (0.72-1.28)	0.795	0.994
COD	Serum uric acid, μmol/L	241/176	1.04 (0.78-1.39)	0.781	0.994
COD	Parathyroid hormone, pg/mL	236/173	0.97 (0.74-1.32)	0.818	0.994
COD	25-hydroxyvitamin D, ng/mL	202/144	1.08 (0.80-1.49)	0.627	0.994
COD	Total carbon dioxide, mmol/L	243/178	1.58 (1.18-2.15)	0.003	0.037*
COD	Serum potassium, mmol/L	256/187	1.02 (0.77-1.35)	0.888	0.994
COD	Glucose, mmol/L	241/177	1.30 (0.95-1.86)	0.123	0.431
COD	Triglycerides, mmol/L	254/186	1.06 (0.81-1.52)	0.697	0.994
COD	Total cholesterol, mmol/L	254/186	0.99 (0.75-1.32)	0.949	0.994
COD	HDL cholesterol, mmol/L	254/186	1.07 (0.81-1.43)	0.637	0.994
COD	LDL cholesterol, mmol/L	254/186	1.00 (0.76-1.33)	0.994	0.994
Carbonate apatite	Serum calcium, mmol/L	244/99	1.06 (0.82-1.37)	0.665	0.716
Carbonate apatite	Albumin-corrected calcium, mmol/L	244/99	1.17 (0.91-1.54)	0.231	0.324
Carbonate apatite	Serum phosphorus, mmol/L	239/98	1.15 (0.89-1.50)	0.288	0.366
Carbonate apatite	Serum magnesium, mmol/L	239/95	0.99 (0.77-1.29)	0.957	0.957
Carbonate apatite	Serum uric acid, μmol/L	241/99	0.85 (0.65-1.10)	0.223	0.324
Carbonate apatite	Parathyroid hormone, pg/mL	236/96	1.32 (1.01-1.79)	0.059	0.118
Carbonate apatite	25-hydroxyvitamin D, ng/mL	202/79	0.75 (0.55-1.01)	0.067	0.118
Carbonate apatite	Total carbon dioxide, mmol/L	243/99	0.77 (0.59-0.99)	0.047	0.118

Stone component	Serum marker	N/events	OR per 1 SD (95% CI)	P value	FDR-adjusted P
Carbonate apatite	Serum potassium, mmol/L	256/101	0.73 (0.56-0.95)	0.022	0.118
Carbonate apatite	Glucose, mmol/L	241/99	0.73 (0.53-0.96)	0.032	0.118
Carbonate apatite	Triglycerides, mmol/L	254/100	0.87 (0.62-1.14)	0.370	0.432
Carbonate apatite	Total cholesterol, mmol/L	254/100	1.30 (1.00-1.69)	0.051	0.118
Carbonate apatite	HDL cholesterol, mmol/L	254/100	1.27 (0.99-1.65)	0.060	0.118
Carbonate apatite	LDL cholesterol, mmol/L	254/100	1.28 (1.00-1.66)	0.056	0.118
Anhydrous uric acid	Serum calcium, mmol/L	244/24	0.71 (0.43-1.13)	0.173	0.395
Anhydrous uric acid	Albumin-corrected calcium, mmol/L	244/24	0.86 (0.52-1.31)	0.542	0.705
Anhydrous uric acid	Serum phosphorus, mmol/L	239/24	1.11 (0.72-1.68)	0.636	0.742
Anhydrous uric acid	Serum magnesium, mmol/L	239/24	0.97 (0.64-1.48)	0.873	0.873
Anhydrous uric acid	Serum uric acid, μmol/L	241/24	1.72 (1.13-2.64)	0.011	0.079
Anhydrous uric acid	Parathyroid hormone, pg/mL	236/21	1.05 (0.63-1.51)	0.807	0.869
Anhydrous uric acid	25-hydroxyvitamin D, ng/mL	202/19	1.32 (0.84-2.00)	0.197	0.395
Anhydrous uric acid	Total carbon dioxide, mmol/L	243/24	0.84 (0.55-1.29)	0.428	0.665
Anhydrous uric acid	Serum potassium, mmol/L	256/25	1.26 (0.84-1.88)	0.254	0.445
Anhydrous uric acid	Glucose, mmol/L	241/23	1.34 (0.92-1.89)	0.105	0.294
Anhydrous uric acid	Triglycerides, mmol/L	254/25	1.84 (1.27-2.84)	0.003	0.048*
Anhydrous uric acid	Total cholesterol, mmol/L	254/25	0.88 (0.55-1.32)	0.554	0.705
Anhydrous uric acid	HDL cholesterol, mmol/L	254/25	0.61 (0.35-0.98)	0.060	0.208
Anhydrous uric acid	LDL cholesterol, mmol/L	254/25	0.55 (0.32-0.90)	0.023	0.108

* FDR-adjusted P<0.05.

Table S7. Final prediction-model specifications and nested internal-validation performance

Stone component	Predictor library	Screen	Algorithm	N/events	OOF ROC AUC (95% CI)	OOF PR AUC	Brier	Log loss	Calibration intercept	Calibration slope
COM	continuous	P<0.20	Random forest (tuned)	256/201	0.697 (0.609-0.785)	0.870	0.146	0.474	0.30	0.80
COD	continuous	none	Elastic net (α=0.50)	256/187	0.654 (0.576-0.732)	0.823	0.180	0.541	-0.32	1.30
Carbonate apatite	continuous	P<0.20	Elastic net (α=0.75)	256/101	0.712 (0.648-0.775)	0.591	0.209	0.605	0.03	1.00

AUC, area under the receiver operating characteristic curve; OOF, out-of-fold; PR, precision–recall; ROC, receiver operating characteristic.
